# Effects of Oral Berry Supplementation on Blood Pressure in Adults with Hypertension or Elevated Blood Pressure: A Systematic Review and Meta-Analysis of Randomized Controlled Trials

**DOI:** 10.3390/nu18101504

**Published:** 2026-05-08

**Authors:** Eduardo Vladimir Guevara Guevara, Sheyla Stefany Gutiérrez Asencios, Ivonne Melanie Gutiérrez Zorrilla, Wilson Valerio Vasquez Rojas, Carlos Vilchez-Perales, Juan Decara, Jamee Guerra Valencia, Akram Hernández-Vásquez, Nataly Dolores Bernuy-Osorio

**Affiliations:** 1 Programa Doctoral en Nutrición, Escuela de Posgrado, Universidad Nacional Agraria La Molina, Lima 15024, Peru; vladimirguevarag@gmail.com (E.V.G.G.); sstefanygutierrez@gmail.com (S.S.G.A.); ivonnegz0426@gmail.com (I.M.G.Z.); ing.valeriovasquez@gmail.com (W.V.V.R.); 2 Departamento de Nutrición, Universidad Nacional Agraria La Molina, Lima 15024, Peru; cvilchezp@lamolina.edu.pe; 3 Instituto de Investigación Biomédica de Málaga y Plataforma en Nanomedicina-IBIMA Plataforma BIONAND, Hospital Universitario Regional de Málaga, UGC Salud Mental, Av. Carlos Haya 82, 29010 Málaga, Spain; juandecara@uma.es; 4 Facultad de Ciencias de la Salud, Universidad Privada del Norte, Lima 15314, Peru; jamee.guerra@upn.pe; 5 Centro de Excelencia en Investigaciones Económicas y Sociales en Salud, Vicerrectorado de Investigación, Universidad San Ignacio de Loyola, Lima 15024, Peru; ahernandez@usil.edu.pe

**Keywords:** hypertension, blood pressure, cardiovascular risk, berry supplementation, anthocyanins, randomized controlled trials, systematic review, meta-analysis

## Abstract

**Background/Objective:** Hypertension is a leading preventable cause of cardiovascular disease, affecting over one billion people worldwide. Berry-derived bioactive compounds may influence vascular function and blood pressure. This systematic review and meta-analysis evaluated the effects of oral berry supplementation on blood pressure in adults with elevated blood pressure or hypertension and additionally assessed its effects on related cardiometabolic outcomes. **Methods:** PubMed, CENTRAL, Embase, and Scopus were searched from inception to May 2025. Eligible studies were randomized controlled trials (RCTs) enrolling adults with hypertension or elevated blood pressure who received oral berry supplementation compared with placebo, standard diet without berries or usual care. Design-specific analytical strategies were employed to preserve within-participant comparisons in cross-over trials. Random-effects meta-analyses were conducted using the restricted maximum likelihood estimator, and the certainty of evidence was assessed using the GRADE framework. PROSPERO registration: CRD420251046489. **Results:** Nine RCTs (547 participants) were included. Berry supplementation did not significantly reduce resting systolic (MD −2.35 mmHg; 95% CI −4.98 to 0.29; *I*^2^ = 65%) or diastolic blood pressure (MD −1.04 mmHg; 95% CI −2.38 to 0.76; *I*^2^ = 60%). A significant reduction in 24 h ambulatory diastolic blood pressure was observed (MD −1.11 mmHg; 95% CI −2.04 to −0.17; *I*^2^ = 0%), driven by cross-over trials. No significant effects were found for vascular, lipid, or inflammatory markers. Certainty of evidence was low to very low across all outcomes. **Conclusions:** Current evidence does not support a consistent effect of berry supplementation on blood pressure or related cardiometabolic outcomes in this population, given the low to very low certainty of the available evidence.

## 1. Introduction

Hypertension is a major global public health concern and the leading preventable risk factor for cardiovascular disease, stroke, and premature mortality [1]. It is estimated to affect more than one billion individuals worldwide, contributing substantially to the global burden of disease [1,2]. Given its chronic nature, effective strategies for the prevention and management of elevated blood pressure remain a critical priority in clinical and public health settings [3].

The management of elevated blood pressure and hypertension involves multiple complementary approaches, including both pharmacological treatment and lifestyle interventions [3]. Current clinical guidelines emphasize that lifestyle modifications are a fundamental component of blood pressure management and are recommended for both the prevention and treatment of elevated blood pressure and hypertension [3,4,5]. These interventions include dietary changes, physical activity, weight management, and other behavioral strategies that contribute to blood pressure control [3]. Among these, dietary modifications represent a key strategy, with healthy dietary patterns such as the Dietary Approaches to Stop Hypertension (DASH) diet consistently recommended to reduce blood pressure levels [3]. These dietary patterns emphasize the consumption of fruits, vegetables, and other nutrient-rich foods. At the same time, research on food-derived bioactive components and their potential health effects has grown substantially over recent decades, particularly in relation to cardiometabolic outcomes [6].

Berries, including fruits such as blueberries, strawberries, cranberries, and others contain bioactive components, including phenolic compounds (such as anthocyanins and flavonols), vitamins, and dietary fiber, which may influence cardiovascular health [7]. The composition and concentration of these compounds can vary across species and are influenced by factors such as genetics, environmental conditions, and processing [7]. These foods have been proposed to exert effects on vascular function, inflammation, and oxidative stress, providing a plausible biological rationale for their potential role in blood pressure regulation [7,8]. These effects may extend beyond blood pressure itself to include vascular and cardiometabolic markers related to blood pressure regulation, providing additional insights into their potential role. Previous systematic reviews and meta-analyses have examined the effects of berry consumption or supplementation on cardiovascular and blood pressure-related outcomes across different study populations [9,10,11,12,13,14].

Although existing systematic reviews and meta-analyses have evaluated the effects of berry consumption or supplementation on blood pressure and some cardiometabolic and vascular markers, several important considerations remain. Previous reviews have often focused on blood pressure outcomes or cardiometabolic and vascular markers [9,10,11,14] rather than synthesizing all of them together and frequently addressing only specific berry types rather than evaluating a broader range of interventions [9,10,11,14], and have included heterogeneous populations, frequently combining healthy individuals with those presenting cardiometabolic conditions [9,10,11,12,13,14]. Even among clinical populations, participants with varying degrees of cardiovascular risk have been grouped together, including those with elevated blood pressure as well as other cardiometabolic conditions [10], which may introduce additional heterogeneity due to differences in baseline risk and associated comorbidities. Furthermore, potential effect modifiers, such as the form of berry supplementation (e.g., whole fruit, juice, or extracts) [9,10,11,14] and duration of intervention [9,14], have not been consistently explored. In addition, the inclusion of both parallel and cross-over randomized controlled trials without clearly accounting for the methodological differences between these designs may affect the estimation and interpretation of pooled effects [9,10,11,14]. Taken together, these factors contribute to variability in the findings and limit the applicability of existing evidence to individuals with elevated blood pressure or hypertension. Therefore, the aim of this systematic review and meta-analysis was to evaluate the effects of oral berry supplementation on blood pressure in adults with elevated blood pressure or hypertension and additionally to assess its effects on related vascular and cardiometabolic outcomes.

## 2. Materials and Methods

### 2.1. Study Design and Protocol Registration

This systematic review and meta-analysis of randomized controlled trials (RCTs) was conducted according to a protocol prospectively registered in the International Prospective Register of Systematic Reviews (PROSPERO) (registration number: CRD420251046489). The review was performed following the methods specified in the registered protocol and is reported in accordance with the Preferred Reporting Items for Systematic Reviews and Meta-Analyses (PRISMA 2020) guidelines [15].

### 2.2. Eligibility Criteria

Eligibility criteria were defined using the PICOS framework (Population, Intervention, Comparator, Outcomes and Study design).

Studies were eligible if they met all of the following criteria:

- Population: adults ≥ 18 years with hypertension or elevated blood pressure, regardless of concomitant antihypertensive treatment.
- Intervention: oral supplementation with berries, including blueberry, cranberry, blackberry, bilberry, strawberry, chokeberry, or tart cherry, administered as whole fruit, juice, powder, standardized extracts, or other derived formulations.
- Comparator: placebo, usual care, or standard diet without berry consumption, regardless of additional dietary recommendations.
- Outcomes: reporting at least one of the main predefined outcomes of interest.
- Study design: RCTs with parallel or cross-over designs.

Studies were excluded if they involved normotensive populations, pregnant women with hypertensive disorders of pregnancy, participants younger than 18 years, non-randomized designs conference abstracts, preprints, studies reporting only descriptive or qualitative data preventing calculation of effect estimates, and publications in languages other than English, Spanish, or Portuguese.

### 2.3. Outcomes of Interest

The outcomes evaluated in this review were based on those pre-specified in the PROSPERO protocol (CRD420251046489). Outcomes were categorized as main outcomes related to blood pressure and additional vascular and cardiometabolic outcomes relevant to hypertension pathophysiology.

#### 2.3.1. Main Outcomes

The primary outcomes of interest were objective measurements of blood pressure, including resting systolic and diastolic blood pressure and 24 h systolic and diastolic blood pressure.

#### 2.3.2. Additional Outcomes

Additional outcomes included vascular and cardiometabolic markers. These comprised arterial stiffness markers, including carotid–femoral pulse wave velocity, brachial–aortic pulse wave velocity, and augmentation index at 75 bpm; endothelial function markers such as flow-mediated dilation; lipid profile parameters including total cholesterol, LDL cholesterol, HDL cholesterol, and triglycerides; and inflammatory biomarkers including CRP and TNF-α.

Some outcomes originally planned in the protocol including mean blood pressure, blood pressure control, antihypertensive medication dose or need, oxidative stress biomarkers, nitric oxide, endothelin-1, interleukin-6, interleukin-1β, MCP-1, adverse events, and adherence to supplementation were not analyzed as they were not reported by the included studies.

### 2.4. Information Sources and Search Strategy

A systematic literature search was conducted in the electronic databases PubMed, Cochrane Central Register of Controlled Trials (CENTRAL), Embase, and Scopus from database inception to the date of the search (11 May 2025), without time restrictions.

The initial search strategy was developed in PubMed using combinations of controlled vocabulary (MeSH) and free-text keywords combined with Boolean operators. The strategy was designed by a biomedical information specialist with experience in systematic reviews and subsequently reviewed and validated by the review team. The final strategy was adapted to the syntax requirements of the remaining databases.

To identify additional relevant studies, reference lists of included studies and relevant reviews were manually screened, and forward citation searching was conducted using snowballing techniques.

The complete search strategies for all electronic databases are provided in Supplementary Material S1.

### 2.5. Study Selection

All records identified through the search were initially imported into EndNote X9 (Clarivate Analytics, Philadelphia, PA, USA) for duplicate removal. The remaining records were then exported in .ris format and imported into the Rayyan web platform to facilitate the screening process.

Study selection was conducted in two sequential stages. First, titles and abstracts of all retrieved records were independently screened by two reviewers (IMGZ and SSGA) using the predefined eligibility criteria. In the second stage, the full texts of potentially eligible articles were independently assessed by the same reviewers, who reapplied the eligibility criteria in detail to determine final study inclusion.

Any disagreements during either stage were resolved through discussion between the reviewers. When consensus could not be reached, a third reviewer (JGV) was consulted to make the final decision.

### 2.6. Data Extraction

Data extraction was performed independently and in duplicate (IMGZ, SSGA, EVGG, and WVVR) using a standardized data extraction form developed in Microsoft Excel 2024 (Microsoft Corporation, Redmond, WA, USA), which was pilot-tested on three studies to ensure consistency before formal data collection. Extracted information included study characteristics (author, year, country, study design), participant characteristics (sample size, age, sex, baseline blood pressure status), intervention details (type of berry, formulation, dose, duration), comparator characteristics, and outcome measurements for each study arm. When necessary, additional data required for quantitative synthesis, such as mean differences, standard deviations, standard errors, or confidence intervals, were derived from the reported data following recommendations from the Cochrane Handbook for Systematic Reviews of Interventions (Chapter 6: Choosing effect measures and computing estimates of effect) [16].

Discrepancies between reviewers during data extraction were resolved through discussion and consensus. A third reviewer (JGV) verified the extracted data and resolved any remaining inconsistencies.

### 2.7. Data Synthesis and Statistical Analysis

A qualitative descriptive synthesis of study characteristics and reported results was initially conducted to assess clinical and methodological heterogeneity among the included studies.

When at least two studies were sufficiently comparable in terms of population, intervention, comparator, and outcomes, quantitative meta-analysis was performed using the meta package in R 4.5.2 (R Foundation for Statistical Computing, Vienna, Austria). Continuous outcomes were summarized as mean differences (MD) with 95% confidence intervals (CI), as the outcomes were measured using comparable units across studies.

Meta-analyses combined evidence from parallel-group and cross-over RCTs. For cross-over trials, treatment effects were estimated using within-participant comparisons, deriving paired mean differences and corresponding standard errors following recommendations from the Cochrane Handbook for Systematic Reviews of Interventions (23.2.6 Methods for incorporating cross-over trials into a meta-analysis; 23.2.7 Approximate analyses of cross-over trials for a meta-analysis; and 23.2.8 Issues in the incorporation of cross-over trials) [16]. When paired standard errors were not reported, they were derived from reported confidence intervals or estimated using the standard deviation of within-participant differences assuming a within-participant correlation coefficient. Because the within-participant correlation was not reported in the included studies, a correlation coefficient of 0.50 was assumed for the primary analyses as a plausible mid-range value as empirical assessments of within-participant correlations in clinical trials suggest median correlations around 0.59 (IQR 0.40–0.81) [17]. Sensitivity analyses were conducted using correlation coefficients of 0.25 and 0.75 to evaluate the robustness of the results to this assumption.

For parallel-group trials, mean differences were calculated as the difference between post-intervention means in the intervention and control groups. Corresponding standard errors were calculated using group-specific standard deviations and sample sizes according to standard formulas for independent groups (Chapter 6: Choosing effect measures and computing estimates of effect) [16].

When trials included multiple intervention arms sharing a single control group, intervention arms were combined into a single intervention group prior to analysis to avoid unit-of-analysis errors, following Cochrane Handbook recommendations (6.5.2.10 Combining groups and 23.3 Studies with more than two intervention groups) [16]. Combined intervention means and standard deviations were calculated using weighted formulas based on sample size before estimating the corresponding effect estimates.

Detailed procedures for the derivation of effect estimates including specific equations, handling of cross-over trials, management of multi-arm studies, and outcome-specific data extraction decisions, including assumptions and data handling procedures, are provided in Supplementary Material S2.

For biochemical outcomes reported using different measurement units across studies (e.g., mg/dL vs. mmol/L), values were converted to common units prior to analysis to ensure comparability across studies.

All effect estimates derived from parallel and cross-over trials were pooled using the generic inverse-variance method. Random-effects meta-analyses were conducted using the restricted maximum likelihood estimator (REML) to estimate between-study variance (τ^2^). Sensitivity analyses were performed using the Hartung–Knapp–Sidik–Jonkman (HKSJ) adjustment to account for uncertainty in the estimation of between-study variance [18]. Statistical heterogeneity was assessed using the *I*^2^ statistic, with values above 50% interpreted as indicating substantial heterogeneity. When heterogeneity was present, potential sources were explored through visual inspection of forest plots and predefined subgroup analyses when sufficient data were available. Subgroup analyses were conducted according to study design (parallel vs. cross-over randomized controlled trials), intervention duration (>8 weeks vs. ≤8 weeks), supplement presentation (freeze-dried powder, capsule, or juice), and berry type.

### 2.8. Risk of Bias Assessment

The risk of bias of the included studies was assessed using the Cochrane Risk of Bias (RoB) tool for randomized controlled trials [19], which evaluates several potential domains of bias: random sequence generation, allocation concealment, blinding of participants and personnel, blinding of outcome assessment, incomplete outcome data, selective reporting, and other sources of bias. Each domain was classified as low risk of bias, unclear risk of bias, or high risk of bias, according to the criteria described in the RoB tool guidance.

The assessment was conducted independently by two reviewers (IMGZ and WVVR), and any disagreements were resolved through discussion and consensus or, when necessary, through consultation with a third reviewer (JGV). The results of the risk of bias assessment were summarized using graphical representations.

### 2.9. Assessment of Reporting Bias

Potential reporting bias was assessed by examining the availability of outcome data across the included studies, including the identification of outcomes that were measured but not reported in the published articles. As all meta-analyses included fewer than 10 studies, formal assessment of publication bias using funnel plots or statistical tests for funnel plot asymmetry was not performed.

### 2.10. Certainty of Evidence Assessment

The certainty of the evidence was assessed using the Grading of Recommendations Assessment, Development and Evaluation (GRADE) approach, considering five domains: risk of bias, inconsistency, indirectness, imprecision, and publication bias. The certainty of evidence for each outcome was rated as high, moderate, low, or very low. Results were summarized in Summary of Findings (SoF) tables using a minimally contextualized approach. Imprecision was evaluated according to the GRADE guidance for minimally contextualized interpretation, considering whether the confidence intervals crossed predefined thresholds of clinical importance and whether the optimal information size (OIS) was achieved [20].

The SoF tables were prepared using GRADEpro GDT software (GRADE Working Group, Hamilton, ON, Canada), and the detailed GRADE evidence profiles are provided in the Supplementary Materials.

### 2.11. Ethical Considerations

As this study was based exclusively on previously published data, ethics committee approval was not required.

## 3. Results

### 3.1. Search Results

**Figure 1:**
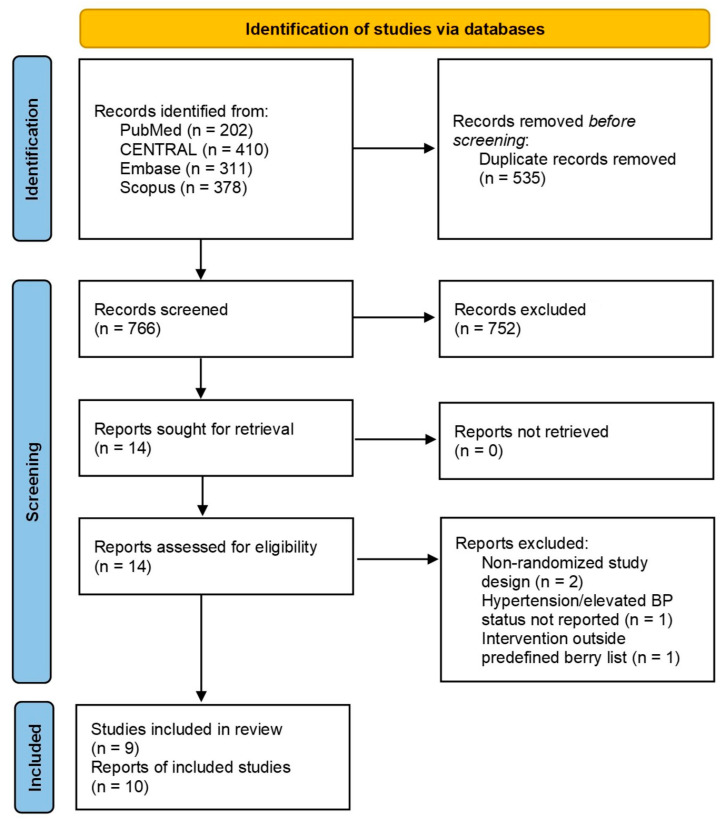
PRISMA 2020 flow diagram of the study selection process.

After duplicate elimination we obtained a total of 766 records, which were screened by title and abstract. A total of 14 reports were assessed for eligibility [21,22,23,24,25,26,27,28,29,30,31,32,33,34]. Finally, 9 RCTs (10 reports) were included (Figure 1). Excluded studies and reasons for exclusion are presented in Supplementary Material S3.

### 3.2. Study Characteristics

A total of nine RCTs involving 547 participants were included in the review. Of these, three studies used a cross-over design [26,28,31], while six were parallel-group randomized trials [22,23,24,27,32,33,34].

The studies were conducted across several countries, including the United States, the United Kingdom, Finland, Norway, South Korea, and Iran. Most trials enrolled adults with elevated blood pressure, prehypertension, or stage 1 hypertension, although specific populations were examined in some studies. Two trials included postmenopausal women, one study included only male participants with early hypertension, and the remaining trials enrolled both men and women. Sample sizes ranged from 15 to 153 participants. All studies evaluated berry-based interventions, administered in different forms such as juices, freeze-dried powders, or capsules containing berry extracts. The berries investigated included chokeberry (*Aronia*), cranberry (*Vaccinium oxycoccus*), tart cherry (*Prunus*), blueberry (*Vaccinium cyanococcus*), and black raspberry (*Rubus*). Control groups generally received placebo beverages, powders, or capsules matched in appearance and macronutrient composition. The duration of the interventions varied across studies, ranging from acute exposure (8 h) to 12 weeks, although most trials lasted 8–12 weeks (Table 1).

### 3.3. Risk of Bias in Studies

**Figure 2:**
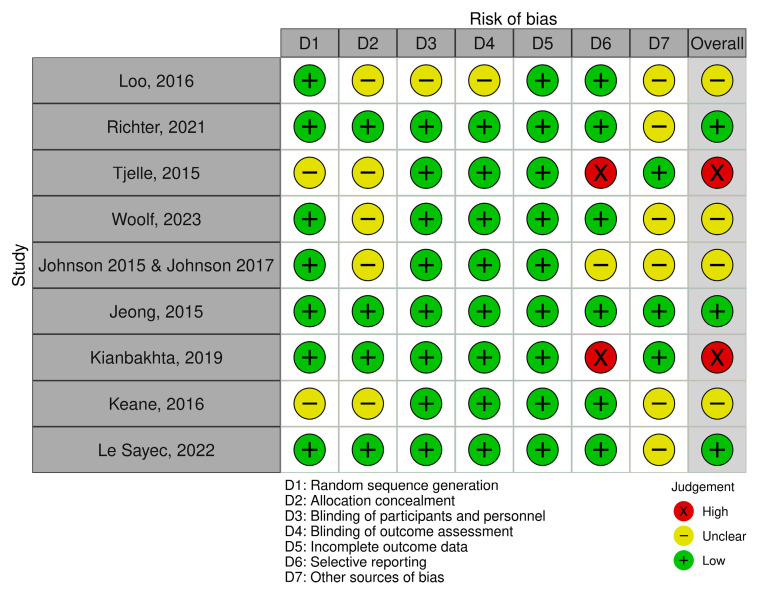
Risk of bias assessment across Cochrane domains for office blood pressure outcomes. Studies included: Loo et al. [28], Richter et al. [31], Tjelle et al. [33], Woolf et al. [34], Johnson et al. [23,24], Jeong et al. [22], Kianbakhta et al. [27], Keane et al. [26], Le Sayec et al. [32].

**Figure 3:**
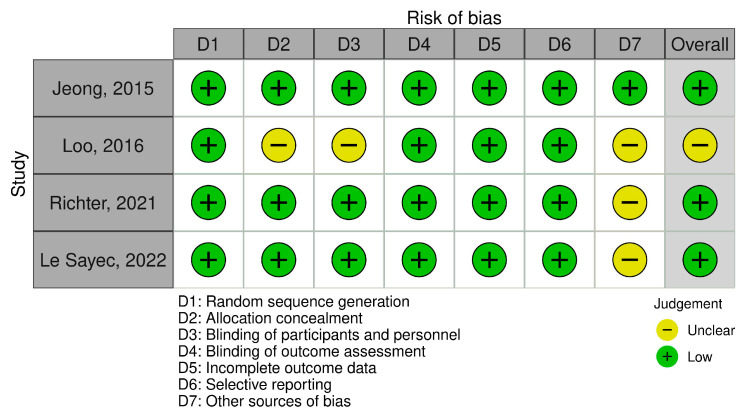
Risk of bias assessment across Cochrane domains for 24 h ambulatory blood pressure outcomes. Studies included: Jeong et al. [22], Loo et al. [28], Richter et al. [31], Le Sayec et al. [32].

**Figure 4:**
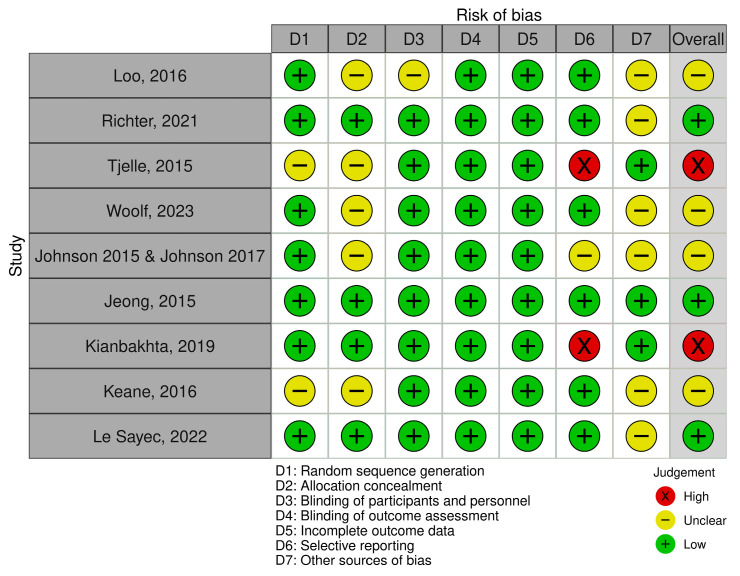
Risk of bias assessment across Cochrane domains for additional vascular and cardiometabolic outcomes. Studies included: Loo et al. [28], Richter et al. [31], Tjelle et al. [33], Woolf et al. [34], Johnson et al. [23,24], Jeong et al. [22], Kianbakhta et al. [27], Keane et al. [26], Le Sayec et al. [32].

Risk of bias was assessed across the Cochrane domains for each outcome evaluated in this review. The corresponding traffic-light plots for office blood pressure, 24 h ambulatory blood pressure, and additional outcomes are presented in Figure 2, Figure 3 and Figure 4.

Overall, most studies were judged as having a low risk of bias in random sequence generation, blinding of participants and personnel, blinding of outcome assessment, and incomplete outcome data. In contrast, allocation concealment was frequently classified as unclear risk of bias. Two trials were judged as having a high risk of bias in selective reporting. For other sources of bias (D7), three studies were rated as low risk, while the remaining trials were classified as unclear risk. The unclear ratings were mainly related to the cross-over design used in three trials, raising concerns about potential carry-over effects, and to industry-related funding where the role of the funder was not fully specified. Overall, most studies were considered to have low or unclear risk of bias.

### 3.4. Main Outcomes: Resting and 24 H Blood Pressure

**Figure 5:**
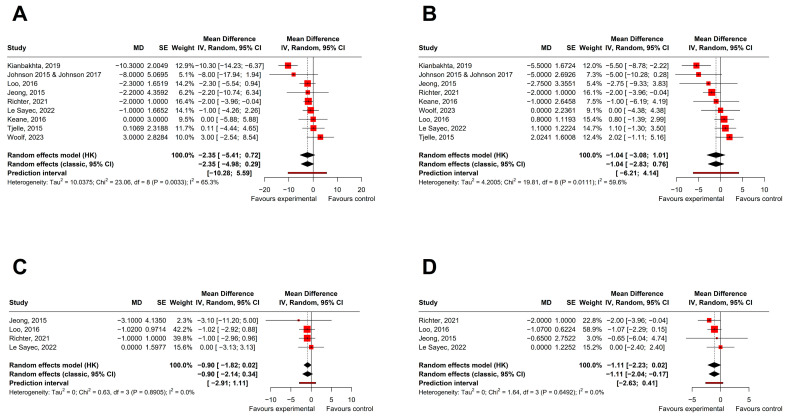
Forest plots of the effects of berry supplementation on resting (office) and 24 h ambulatory systolic and diastolic blood pressure, stratified by study design (parallel vs. cross-over randomized controlled trials). (**A**) Resting systolic blood pressure [22,23,24,26,27,28,31,32,33,34]; (**B**) resting diastolic blood pressure [22,23,24,26,27,28,31,32,33,34]; (**C**) 24 h ambulatory systolic blood pressure [22,28,31,32]; (**D**) 24 h ambulatory diastolic blood pressure [22,28,31,32]. For Jeong, 2015 experimental arms were combined. For Keane, 2016 [26] and Richter, 2021 [31], effect calculated assuming r = 0.5 (cross-over). Red squares represent individual study effect estimates, with the center indicating the point estimate and the size proportional to study weight. Horizontal lines indicate 95% confidence intervals. Black diamonds represent pooled estimates from random-effects models using Hartung–Knapp–Sidik–Jonkman (upper) and REML (lower), with the center indicating the pooled estimate and the width representing the 95% confidence interval. The vertical solid line represents the line of no effect (MD = 0), and vertical dashed lines indicate pooled estimates from each model. CI, confidence interval; *I*^2^, heterogeneity; Tau^2^, between-study variance.

The results for the main outcomes are summarized in Table 2, Table 3, Table 4 and Table 5 and presented in the corresponding forest plots. These outcomes included resting (office) systolic (Figure 5A) and diastolic blood pressure (Figure 5B) and 24 h ambulatory systolic (Figure 5C) and diastolic blood pressure (Figure 5D). To explore potential methodological differences between trials, analyses were additionally stratified according to study design (parallel vs. cross-over trials).

### 3.5. Resting Systolic Blood Pressure

Nine randomized trials including 547 participants evaluated the effect of berry interventions on resting systolic blood pressure [22,23,24,26,27,28,31,32,33,34]. The overall pooled estimate combining parallel and cross-over trials showed no statistically significant effect (MD −2.35 mmHg, 95% CI −4.98 to 0.29, *p* = 0.081; *I*^2^ = 65.3%). When analyzed by study design, the six parallel-group trials (n = 455) showed a non-significant reduction in systolic blood pressure (MD −2.85 mmHg, 95% CI −7.16 to 1.46, *p* = 0.195; *I*^2^ = 77.3%). In contrast, the three cross-over trials (n = 92) showed a significant reduction in systolic blood pressure (MD −1.92 mmHg, 95% CI −3.54 to −0.31, *p* = 0.019; *I*^2^ = 0%) (Table 2).

Subgroup analyses suggested that interventions lasting ≤8 weeks were associated with a significant reduction in systolic blood pressure (MD −2.08 mmHg, 95% CI −3.65 to −0.52, *p* = 0.009), whereas studies lasting >8 weeks did not show a significant effect (MD −2.20 mmHg, 95% CI −7.83 to 3.43, *p* = 0.443).

**Table 2:** Systolic blood pressure (mmHg).

Group/Subgroup	Analysis Specification	Studies (k)	Participants (N)	RE Method	Mean Difference (95% CI)	*p*-Value	*I* ^2^
Overall	Parallel RCT + Cross-over RCT (r = 0.50)	9 [22,23,24,26,27,28,31,32,33,34]	547	REML + HKSJ	−2.35 (−5.41, 0.72)	0.115	65.3
				REML + classic	−2.35 (−4.98, 0.29)	0.081	65.3
	Parallel RCT	6 [22,23,24,27,32,33,34]	455	REML + HKSJ	−2.85 (−8.26, 2.57)	0.234	77.3
				REML + classic	−2.85 (−7.16, 1.46)	0.195	77.3
	Cross-over RCT (r = 0.50)	3 [26,28,31]	92	REML + HKSJ	−1.92 (−3.64, −0.21)	0.040	0.0
				REML + classic	−1.92 (−3.54, −0.31)	0.019	0.0
	Cross-over RCT (r = 0.25)	3 [26,28,31]	92	REML + HKSJ	−1.97 (−3.62, −0.31)	0.036	0.0
				REML + classic	−1.97 (−3.83, −0.10)	0.039	0.0
	Cross-over RCT (r = 0.75)	3 [26,28,31]	92	REML + HKSJ	−1.87 (−3.64, −0.10)	0.045	0.0
				REML + classic	−1.87 (−3.09, −0.65)	0.003	0.0
Subgroup: Duration	>8 weeks (Parallel RCT + Cross-over RCT (r = 0.50))	4 [27,32,33,34]	370	REML + HKSJ	−2.20 (−11.34, 6.94)	0.499	85.7
				REML + classic	−2.20 (−7.83, 3.43)	0.443	85.7
	≤8 weeks (Parallel RCT + Cross-over RCT (r = 0.50))	5 [22,23,24,26,28,31]	177	REML + HKSJ	−2.08 (−3.60, −0.57)	0.019	0.0
				REML + classic	−2.08 (−3.65, −0.52)	0.009	0.0
Subgroup: Supplement presentation	Freeze-dried powder (Parallel RCT + Cross-over RCT (r = 0.50))	2 [23,24,34]	83	REML + HKSJ	−1.70 (−70.83, 67.44)	0.808	72.1
				REML + classic	−1.70 (−12.36, 8.97)	0.755	72.1
	Capsule (Parallel RCT + Cross-over RCT (r = 0.50))	3 [22,27,32]	242	REML + HKSJ	−4.74 (−17.81, 8.33)	0.259	84.7
				REML + classic	−4.74 (−11.03, 1.55)	0.140	84.7
	Juice (Parallel RCT + Cross-over RCT (r = 0.50))	4 [26,28,31,33]	222	REML + HKSJ	−1.70 (−3.22, −0.17)	0.039	0.0
				REML + classic	−1.70 (−3.22, −0.18)	0.029	0.0
Subgroup: Berry type	Blueberry	2 [23,24,34]	83	REML + HKSJ	−1.70 (−70.83, 67.44)	0.808	72.1
				REML + classic	−1.70 (−12.36, 8.97)	0.755	72.1
	Caucasian whortleberry	1 [27]	100	REML + HKSJ	−10.30 (−14.23, −6.37)	<0.001	NA
				REML + classic	−10.30 (−14.23, −6.37)	<0.001	NA
	Chokeberry	3 [28,32,33]	264	REML + HKSJ	−1.30 (−4.08, 1.49)	0.184	0.0
				REML + classic	−1.30 (−3.35, 0.75)	0.216	0.0
	Cranberry	1 [31]	40	REML + HKSJ	−2.00 (−3.96, −0.04)	0.046	NA
				REML + classic	−2.00 (−3.96, −0.04)	0.046	NA
	Black raspberry	1 [22]	45	REML + HKSJ	−2.20 (−10.74, 6.34)	0.614	NA
				REML + classic	−2.20 (−10.74, 6.34)	0.614	NA
	Montmorency tart cherry	1 [26]	15	REML + HKSJ	0.00 (−5.88, 5.88)	1.000	NA
				REML + classic	0.00 (−5.88, 5.88)	1.000	NA

REML: Restricted Maximum Likelihood; HKSJ: Hartung–Knapp–Sidik–Jonkman; NA: Not applicable; for parallel RCT with two intervention arms compilation in one single arm was performed as recommended by Cochrane manual (Chapter 6; 6.5.2.10) using sd: √[[(n1−1)·sd1^2^ + (n2−1)·sd2^2^ + (n1·n2/(n1 + n2))·(mean1^2^ + mean2^2^ − 2·mean1·mean2)]/(n1 + n2−1)]; mean difference was calculated for cross-over RCT using correlation coefficients 0.5, 0.25 and 0.75 as recommended by Cochrane manual (Chapter 6; 6.5.2.10) using (sd_treat^2^ + sd_cont^2^ − 2·r·sd_treat·sd_cont); parallel RCT with 3 arms: Tjelle 2015 [33] and Jeong, 2015 [22].

### 3.6. Resting Diastolic Blood Pressure

Nine trials including 547 participants assessed the effects of berry interventions on resting diastolic blood pressure [22,23,24,26,27,28,31,32,33,34]. The overall pooled estimate showed no statistically significant effect (MD −1.04 mmHg, 95% CI −2.38 to 0.76, *p* = 0.258; *I*^2^ = 59.6%). Similarly, the parallel-group trials (n = 455) showed no significant reduction in diastolic blood pressure (MD −1.37 mmHg, 95% CI −4.15 to 1.40, *p* = 0.332; *I*^2^ = 69.4%). The cross-over trials (n = 92) also showed no significant effect (MD −0.71 mmHg, 95% CI −2.85 to 1.43, *p* = 0.515; *I*^2^ = 42.7%) (Table 3).

**Table 3:** Diastolic blood pressure (mmHg).

Group/Subgroup	Analysis Specification	Studies (k)	Participants (N)	RE Method	Mean Difference (95% CI)	*p*-Value	*I* ^2^
Overall	Parallel RCT + Cross-over RCT (r = 0.50)	9 [22,23,24,26,27,28,31,32,33,34]	547	REML + HKSJ	−1.04 (−3.08, 1.01)	0.277	59.6
				REML + classic	−1.04 (−2.83, 0.76)	0.258	59.6
	Parallel RCT	6 [22,23,24,27,32,33,34]	455	REML + HKSJ	−1.37 (−4.84, 2.09)	0.355	69.4
				REML + classic	−1.37 (−4.15, 1.40)	0.332	69.4
	Cross-over RCT (r = 0.50)	3 [26,28,31]	92	REML + HKSJ	−0.71 (−4.68, 3.26)	0.522	42.7
				REML + classic	−0.71 (−2.85, 1.43)	0.515	42.7
	Cross-over RCT (r = 0.25)	3 [26,28,31]	92	REML + HKSJ	−0.59 (−4.63, 3.44)	0.592	30.4
				REML + classic	−0.59 (−2.83, 1.65)	0.604	30.4
	Cross-over RCT (r = 0.75)	3 [26,28,31]	92	REML + HKSJ	−0.84 (−2.81, 1.12)	0.400	55.4
				REML + classic	−0.84 (−2.81, 1.12)	0.400	55.4
Subgroup: Duration	>8 weeks (Parallel RCT + Cross-over RCT (r = 0.50))	4 [27,32,33,34]	370	REML + HKSJ	−0.55 (−5.96, 4.86)	0.767	77.1
				REML + classic	−0.55 (−3.92, 2.82)	0.748	77.1
	≤8 weeks (Parallel RCT + Cross-over RCT (r = 0.50))	5 [22,23,24,26,28,31]	177	REML + HKSJ	−1.35 (−3.90, 1.19)	0.214	33.7
				REML + classic	−1.35 (−3.34, 0.63)	0.182	33.7
Subgroup: Supplement presentation	Freeze-dried powder (Parallel RCT + Cross-over RCT (r = 0.50))	2 [23,24,34]	83	REML + HKSJ	−2.27 (−33.91, 29.36)	0.529	51.0
				REML + classic	−2.27 (−7.16, 2.61)	0.361	51.0
	Capsule (Parallel RCT + Cross-over RCT (r = 0.50))	3 [22,27,32]	242	REML + HKSJ	−2.20 (−11.06, 6.66)	0.398	80.7
				REML + classic	−2.20 (−6.60, 2.20)	0.328	80.7
	Juice (Parallel RCT + Cross-over RCT (r = 0.50))	4 [26,28,31,33]	222	REML + HKSJ	−0.09 (−3.06, 2.88)	0.930	50.3
				REML + classic	−0.09 (−2.09, 1.91)	0.930	50.3
Subgroup: Berry type	Blueberry	2 [23,24,34]	83	REML + HKSJ	−2.27 (−33.91, 29.36)	0.529	51.0
				REML + classic	−2.27 (−7.16, 2.61)	0.361	51.0
	Caucasian whortleberry	1 [27]	100	REML + HKSJ	−5.50 (−8.78, −2.22)	0.001	NA
				REML + classic	−5.50 (−8.78, −2.22)	0.001	NA
	Chokeberry	3 [28,32,33]	264	REML + HKSJ	1.17 (−0.24, 2.57)	0.071	0.0
				REML + classic	1.17 (−0.27, 2.60)	0.112	0.0
	Cranberry	1 [31]	40	REML + HKSJ	−2.00 (−3.96, −0.04)	0.046	NA
				REML + classic	−2.00 (−3.96, −0.04)	0.046	NA
	Black raspberry	1 [22]	45	REML + HKSJ	−2.75 (−9.33, 3.83)	0.412	NA
				REML + classic	−2.75 (−9.33, 3.83)	0.412	NA
	Montmorency tart cherry	1 [26]	15	REML + HKSJ	−1.00 (−6.19, 4.19)	0.705	NA
				REML + classic	−1.00 (−6.19, 4.19)	0.705	NA

REML: Restricted Maximum Likelihood; HKSJ: Hartung–Knapp–Sidik–Jonkman; NA: Not applicable; for parallel RCT with two intervention arms compilation in one single arm was performed as recommended by Cochrane manual (Chapter 6; 6.5.2.10) using sd: √[[(n1−1)·sd1^2^ + (n2−1)·sd2^2^ + (n1·n2/(n1 + n2))·(mean1^2^ + mean2^2^ − 2·mean1·mean2)]/(n1 + n2−1)]; mean difference was calculated for cross-over RCT using correlation coefficients 0.5, 0.25 and 0.75 as recommended by Cochrane manual (Chapter 6; 6.5.2.10) using (sd_treat^2^ + sd_cont^2^ − 2·r·sd_treat·sd_cont); parallel RCT with 3 arms: Tjelle 2015 [33] and Jeong, 2015 [22].

Subgroup analyses according to intervention duration, supplement presentation, and berry type did not reveal consistent differences across studies.

### 3.7. 24 H Ambulatory Systolic Blood Pressure

Four trials including 219 participants evaluated the effect of berry interventions on 24 h systolic blood pressure [22,28,31,32]. The pooled estimate showed no statistically significant reduction (MD −0.90 mmHg, 95% CI −2.14 to 0.34, *p* = 0.153; *I*^2^ = 0%). Parallel-group trials (n = 142) showed no significant effect (MD −0.40 mmHg, 95% CI −3.32 to 2.52, *p* = 0.787), and cross-over trials (n = 77) also did not show a significant reduction in systolic blood pressure (MD −1.07 mmHg, 95% CI −2.41 to 0.28, *p* = 0.120) (Table 4).

**Table 4:** 24 h Systolic blood pressure (mmHg).

Group/Subgroup	Analysis Specification	Studies (k)	Participants (N)	RE Method	Mean Difference (95% CI)	*p*-Value	*I* ^2^
Overall	Parallel RCT + Cross-over RCT	4 [22,28,31,32]	219	REML + HKSJ	−0.90 (−1.82, 0.02)	0.052	0.0
				REML + classic	−0.90 (−2.14, 0.34)	0.153	0.0
	Parallel RCT	2 [22,32]	142	REML + HKSJ	−0.40 (−13.65, 12.84)	0.765	0.0
				REML + classic	−0.40 (−3.32, 2.52)	0.787	0.0
	Cross-over RCT (r = 0.50)	2 [28,32]	77	REML + HKSJ	−1.07 (−2.11, −0.03)	0.048	0.0
				REML + classic	−1.07 (−2.41, 0.28)	0.120	0.0
	Cross-over RCT (r = 0.25)	2 [28,32]	77	REML + HKSJ	−1.01 (−1.14, −0.89)	0.006	0.0
				REML + classic	−1.01 (−2.50, 0.48)	0.184	0.0
	Cross-over RCT (r = 0.75)	2 [28,32]	77	REML + HKSJ	−1.01 (−1.13, −0.89)	0.006	0.0
				REML + classic	−1.01 (−2.13, 0.11)	0.078	0.0
Subgroup: Duration	>8 weeks (Parallel RCT + Cross-over RCT)	1 [32]	97	REML + HKSJ	0.00 (−3.13, 3.13)	1.000	NA
				REML + classic	0.00 (−3.13, 3.13)	1.000	NA
	≤8 weeks (Parallel RCT + Cross-over RCT)	3 [22,28,31]	122	REML + HKSJ	−1.08 (−2.19, 0.04)	0.053	0.0
				REML + classic	−1.08 (−2.52, 0.37)	0.143	0.0
Subgroup: Supplement presentation	Capsule (Parallel RCT + Cross-over RCT)	2 [22,32]	142	REML + HKSJ	−0.40 (−13.65, 12.84)	0.765	0.0
				REML + classic	−0.40 (−3.32, 2.52)	0.787	0.0
	Juice (Parallel RCT + Cross-over RCT)	2 [28,31]	77	REML + HKSJ	−1.01 (−1.14, −0.88)	0.006	0.0
				REML + classic	−1.01 (−2.38, −0.36)	0.147	0.0
Subgroup: Berry type	Chokeberry	2 [28,32]	134	REML + HKSJ	−0.74 (−6.50, 5.01)	0.348	0.0
				REML + classic	−0.74 (−2.37, 0.88)	0.370	0.0
	Cranberry	1 [31]	40	REML + HKSJ	−1.00 (−2.96, 0.96)	0.317	NA
				REML + classic	−1.00 (−2.96, 0.96)	0.317	NA
	Black raspberry	1 [22]	45	REML + HKSJ	−3.10 (−11.20, 5.00)	0.453	NA
				REML + classic	−3.10 (−11.20, 5.00)	0.453	NA

REML: Restricted Maximum Likelihood; HKSJ: Hartung–Knapp–Sidik–Jonkman; NA: Not applicable; for parallel RCT with two intervention arms compilation in one single arm was performed as recommended by Cochrane manual (Chapter 6; 6.5.2.10) using sd: √[[(n1−1)·sd1^2^ + (n2−1)·sd2^2^ + (n1·n2/(n1 + n2))·(mean1^2^ + mean2^2^ − 2·mean1·mean2)]/(n1 + n2−1)]; mean difference was calculated for cross-over RCT using correlation coefficients 0.5, 0.25 and 0.75 as recommended by Cochrane manual (Chapter 6; 6.5.2.10) using (sd_treat^2^ + sd_cont^2^ − 2·r·sd_treat·sd_cont); parallel RCT with 3 arms: Jeong, 2015 [22].

### 3.8. 24 H Ambulatory Diastolic Blood Pressure

Four trials including 219 participants assessed 24 h diastolic blood pressure [22,28,31,32]. The overall pooled estimate showed a statistically significant effect (MD −1.11 mmHg, 95% CI −2.04 to −0.17, *p* = 0.020; *I*^2^ = 0%). Parallel-group trials (n = 142) showed no significant difference (MD −0.11 mmHg, 95% CI −2.30 to 2.09, *p* = 0.924), whereas cross-over trials (n = 77) showed a significant effect (MD −1.33 mmHg, 95% CI −2.37 to −0.29, *p* = 0.012) (Table 5).

Sensitivity analyses using the HKSJ adjustment resulted in wider confidence intervals, and statistical significance differed in some analyses.

**Table 5:** 24 h diastolic blood pressure (mmHg).

Group/Subgroup	Analysis Specification	Studies (k)	Participants (N)	RE Method	Mean Difference (95% CI)	*p*-Value	*I* ^2^
Overall	Parallel RCT + Cross-over RCT	4 [22,28,31,32]	219	REML + HKSJ	−1.11 (−2.23, 0.02)	0.052	0.0
				REML + classic	−1.11 (−2.04, −0.17)	0.021	0.0
	Parallel RCT	2 [22,32]	142	REML + HKSJ	−0.11 (−3.18, 2.96)	0.733	0.0
				REML + classic	−0.11 (−2.30, 2.09)	0.923	0.0
	Cross-over RCT (r = 0.50)	2 [28,31]	77	REML + HKSJ	−1.33 (−6.63, 3.97)	0.194	0.0
				REML + classic	−1.33 (−2.37, −0.29)	0.012	0.0
	Cross-over RCT (r = 0.25)	2 [28,31]	77	REML + HKSJ	−1.26 (−6.03, 3.51)	0.184	0.0
				REML + classic	−1.26 (−2.35, −0.17)	0.023	0.0
	Cross-over RCT (r = 0.75)	2 [28,31]	77	REML + HKSJ	−1.48 (−7.34, 4.38)	0.193	0.0
				REML + classic	−1.48 (−2.39, −0.56)	0.002	0.0
Subgroup: Duration	>8 weeks (Parallel RCT + Cross-over RCT)	1 [32]	97	REML + HKSJ	0.00 (−2.40, 2.40)	1.000	NA
				REML + classic	0.00 (−2.40, 2.40)	1.000	NA
	≤8 weeks (Parallel RCT + Cross-over RCT)	3 [22,28,31]	122	REML + HKSJ	−1.31 (−2.61, −0.00)	0.050	0.0
				REML + classic	−1.31 (−2.32, −0.29)	0.012	0.0
Subgroup: Supplement presentation	Capsule (Parallel RCT + Cross-over RCT)	2 [22,32]	142	REML + HKSJ	−0.11 (−3.18, 2.96)	0.733	0.0
				REML + classic	−0.11 (−2.30, 2.09)	0.924	0.0
	Juice (Parallel RCT + Cross-over RCT)	2 [28,31]	77	REML + HKSJ	−1.33 (−6.63, 3.97)	0.194	0.0
				REML + classic	−1.33 (−2.37, −0.29)	0.012	0.0
Subgroup: Berry type	Chokeberry	2 [28,32]	134	REML + HKSJ	−0.85 (−6.34, 4.64)	0.299	0.0
				REML + classic	−0.85 (−1.94, 0.24)	0.125	0.0
	Cranberry	1 [31]	40	REML + HKSJ	−2.00 (−3.96, −0.04)	0.045	NA
				REML + classic	−2.00 (−3.96, −0.04)	0.045	NA
	Black raspberry	1 [22]	45	REML + HKSJ	−0.65 (−6.04, 4.74)	0.813	NA
				REML + classic	−0.65 (−6.04, 4.74)	0.813	NA

REML: Restricted Maximum Likelihood; HKSJ: Hartung–Knapp–Sidik–Jonkman; NA: Not applicable; for parallel RCT with two intervention arms compilation in one single arm was performed as recommended by Cochrane manual (Chapter 6; 6.5.2.10) using sd: √[[(n1−1)·sd1^2^ + (n2−1)·sd2^2^ + (n1·n2/(n1 + n2))·(mean1^2^ + mean2^2^ − 2·mean1·mean2)]/(n1 + n2−1)]; mean difference was calculated for cross-over RCT using correlation coefficients 0.5, 0.25 and 0.75 as recommended by Cochrane manual (Chapter 6; 6.5.2.10) using (sd_treat^2^ + sd_cont^2^ − 2·r·sd_treat·sd_cont); parallel RCT with 3 arms: Jeong, 2015 [22].

### 3.9. Additional Outcomes: Vascular Function, Lipid Profile, and Inflammatory Markers

The results for additional outcomes, including vascular function, lipid profile, and inflammatory markers, are summarized narratively in the text, with detailed estimates provided in Supplementary Tables S1–S3 and the corresponding forest plots (Supplementary Figures S1–S10).

#### 3.9.1. Vascular Function Markers

Across vascular function markers, no consistent or statistically significant effects of berry supplementation were observed. Augmentation index (AIx75) [31,32,34], flow-mediated dilation (FMD) [32,34], and pulse wave velocity (both carotid–femoral and brachial–aortic) [23,24,31,32,34] generally showed small and non-significant differences compared with control groups (Supplementary Table S1).

#### 3.9.2. Lipid Profile

No significant effects were observed for lipid profile outcomes, including total cholesterol [28,31,32,34], HDL cholesterol [28,31,32,34], LDL cholesterol [31,32,34], and triglycerides. Effect estimates were generally small and not statistically significant across studies [28,31,32,34] (Supplementary Table S2).

#### 3.9.3. Inflammatory Markers

For inflammatory markers, no significant effects were observed for CRP [22,23,24,31] or TNF-α [23,24,28]. Estimates were imprecise across studies (Supplementary Table S3).

### 3.10. Reporting Bias

Due to the limited number of studies (<10), formal assessment of publication bias using funnel plots or Egger’s test was not considered reliable.

### 3.11. Certainty of Evidence

SoF tables for the main outcomes are presented in Table 6. For additional outcomes, SoF tables and the corresponding GRADE evidence profiles are provided in the Supplementary Material (Supplementary Tables S4–S6).

The certainty of evidence for the main outcomes ranged from low to very low. Evidence for resting systolic and diastolic blood pressure was rated as very low certainty, mainly due to concerns regarding risk of bias, heterogeneity across studies, and potential publication bias related to industry funding. In addition, imprecision contributed to further downgrading for systolic blood pressure.

In contrast, the certainty of evidence for 24 h systolic and diastolic blood pressure was rated as low, primarily due to imprecision and potential publication bias, while no important heterogeneity or major concerns regarding risk of bias were identified for these outcomes.

For the additional outcomes, the certainty of evidence ranged from low to very low. Evidence was rated as low certainty for augmentation index at 75 bpm, carotid–femoral pulse wave velocity, LDL cholesterol, and CRP. For these outcomes, downgrading was mainly driven by imprecision, as the optimal information size was not reached, together with concerns regarding potential publication bias related to industry funding.

Evidence was rated as very low certainty for flow-mediated dilation, brachial–aortic pulse wave velocity, total cholesterol, HDL cholesterol, triglycerides, and TNF-α. In these outcomes, downgrading resulted from combinations of risk of bias, heterogeneity across studies, and imprecision, which in some cases was considered very serious, particularly in analyses with a small number of studies.

## 4. Discussion

The aim of this systematic review and meta-analysis was to evaluate the effects of berry supplementation on blood pressure and related vascular and cardiometabolic outcomes in adults with elevated blood pressure or hypertension. Overall, berry supplementation was not associated with a statistically significant reduction in resting or 24 h systolic or diastolic blood pressure. Although a statistically significant reduction was observed for 24 h diastolic blood pressure, the certainty of the evidence for this outcome was low. Across all blood pressure outcomes, the estimated effects were small in magnitude, with reductions generally below 3 mmHg, reflecting small observed effect sizes; however, given the low to very low certainty of the evidence, the clinical relevance of these findings remains uncertain. Similarly, no statistically significant effects were observed for additional outcomes, including vascular function markers, lipid profile, and inflammatory biomarkers. The certainty of the evidence ranged from low to very low across all outcomes, mainly due to imprecision, heterogeneity, and concerns regarding risk of bias. Overall, the current evidence does not provide sufficient evidence to support an effect of berry supplementation on blood pressure or related cardiometabolic outcomes in this population.

Previous systematic reviews have addressed the effects of specific types of berries and often included mixed populations, combining healthy individuals with those presenting cardiometabolic conditions [9,10,11,14]. In contrast, the present systematic review focused specifically on individuals with elevated blood pressure or hypertension. Additionally, methodological differences may contribute to variations across findings. Some previous meta-analyses included both parallel and cross-over trials without clearly describing design-specific analytical approaches [10,11], and in some cases, cross-over trials appear to have been analyzed using methods typically applied to parallel designs [10,11,14]. In contrast, the present systematic review applied design-specific analytical strategies, preserving within-participant comparisons in cross-over trials. Furthermore, prior systematic reviews often did not explore relevant sources of heterogeneity, such as the form of berry supplementation [9,10,11,14] or intervention duration [9,14], which were considered in the present analysis.

In line with our findings, a systematic review and meta-analysis conducted by Delpino et al., which included participants with cardiometabolic diseases, reported no significant reductions in systolic or diastolic blood pressure, with pooled estimates of −0.81 mmHg (95% CI: −2.26, 0.63) for systolic blood pressure and −0.15 mmHg (95% CI: −1.36, 1.05) for diastolic blood pressure [10]. These findings are consistent in direction, with effect estimates close to the null and without statistically significant differences. However, it is important to note that the population included by Delpino et al. was more heterogeneous, as it encompassed individuals with a range of cardiometabolic conditions without requiring elevated blood pressure or hypertension as an inclusion criterion. Although their pooled estimates appeared more precise, the individual trials included in that review were also characterized by relatively small sample sizes, generally not exceeding 90 participants, a limitation that is similarly observed in the trials included in the present review. Similar findings have been reported in earlier systematic reviews and meta-analyses focusing on specific berry types. For example, Zhu et al. found no significant effect of blueberry supplementation on systolic or diastolic blood pressure, with pooled estimates close to the null [14], while Eslami et al. reported no significant reductions in blood pressure following cherry supplementation [11]. In contrast, Carvalho et al. observed a significant reduction in diastolic blood pressure following blueberry supplementation [9]. In the present review, subgroup analyses by berry type identified statistically significant effects for certain outcomes and specific berries (e.g., Caucasian whortleberry and cranberry); however, these findings were based on single trials and were not consistently observed across studies or outcomes. These findings should therefore be interpreted with caution, as they may reflect isolated findings rather than consistent effects across berry types. These methodological differences may contribute to variability in effect estimates and limit direct comparability with the present review.

Evidence for other vascular and cardiometabolic outcomes appears to be more heterogeneous. Carvalho et al. reported significant reductions in total and LDL cholesterol following blueberry supplementation, with moderate certainty of evidence [9], and similar findings have been described by Huang et al. for lipid-related outcomes [13]. Consistent with this pattern, another systematic review including mixed populations has also reported improvements in cardiovascular risk markers following berry consumption [35]. In addition, Deng et al. reported significant improvements in endothelial function, as measured by flow-mediated dilation, following blueberry supplementation [36], although this analysis was limited to a specific berry type and included heterogeneous populations. Likewise, Norouzzadeh et al. found significant reductions in CRP with moderate certainty, although the evidence for other inflammatory markers such as TNF-α was of lower certainty [37]. However, these findings should be interpreted with caution, as these analyses generally included mixed populations and were not restricted to individuals with elevated blood pressure or hypertension. These differences in population characteristics and outcome focus may limit direct comparability with the present review.

Taken together, the available evidence suggests that while small effects may be observed for certain cardiometabolic outcomes, these findings are not consistently replicated for blood pressure and are less evident in analyses restricted to clinically relevant populations, such as individuals with elevated blood pressure or hypertension. Regarding blood pressure outcomes, it is important to note that these comparisons are based on resting measurements, as previous meta-analyses did not evaluate 24 h ambulatory blood pressure. Therefore, the findings related to 24-hour blood pressure in the present review represent additional evidence that is not directly comparable with prior studies.

Subgroup analyses suggested the presence of heterogeneity in the effects of berry supplementation across studies, particularly for blood pressure outcomes according to study design and intervention duration. Differences by study design were observed for resting systolic blood pressure, with cross-over trials showing statistically significant reductions, whereas parallel-group trials did not demonstrate significant effects. While these findings may reflect differences in study characteristics, they should be interpreted with caution given the limited number of studies and the exploratory nature of subgroup analyses. Similarly, analyses according to intervention duration suggested that shorter interventions (≤8 weeks) were associated with reductions in specific blood pressure outcomes, particularly in resting systolic blood pressure and 24 h diastolic blood pressure, whereas longer interventions (>8 weeks) did not show significant effects. This pattern may indicate transient physiological responses; however, it should be interpreted with caution, as it may also reflect heterogeneity in study design, populations, adherence, and intervention characteristics. In particular, shorter-duration subgroups in these analyses included a higher proportion of cross-over trials, which may provide more precise estimates due to within-participant comparisons. Therefore, a true attenuation of effect over time cannot be conclusively established. For additional outcomes, subgroup analyses according to study design were also performed; however, no consistent differences were observed between cross-over and parallel trials across these outcomes. Overall, these findings highlight variability in the observed effects across studies and suggest that the impact of berry supplementation may depend on specific study and intervention characteristics. However, these observations should be considered hypothesis-generating rather than confirmatory.

The overall certainty of the evidence was rated as low to very low across most outcomes, including the main outcomes of resting SBP and DBP. This was primarily driven by imprecision, as reflected by wide confidence intervals, small sample sizes, and a limited number of studies for several outcomes, in some cases based on only two trials. Most of the included trials enrolled a limited number of participants, which reduces the ability to detect small but potentially relevant effects. For the main outcomes, additional concerns related to risk of bias were identified in several studies, particularly regarding blinding procedures and incomplete outcome reporting, which may have influenced the observed effect estimates. Taken together, these factors indicate that the current evidence base is limited and that the true effect of berry supplementation on blood pressure and related outcomes remains uncertain.

This review has several limitations that should be considered. First, the number of available trials was limited for several outcomes, particularly for the 24 h blood pressure outcomes and all additional outcomes, including vascular, lipid, and inflammatory markers, with some analyses based on only a small number of studies. This restricted the ability to perform more robust quantitative syntheses and limited the exploration of potential sources of variability across studies. Second, clinical heterogeneity across studies should be considered. The included trials evaluated different types of berries and formulations, with an unequal distribution of evidence across interventions. Although subgroup analyses were conducted for key outcomes, these were often based on a limited number of studies and small sample sizes, restricting the ability to draw firm conclusions regarding potential differences by berry type or form of administration. Similarly, variability in baseline blood pressure levels across studies may have contributed to heterogeneity in the observed treatment effects, as differences in initial blood pressure levels can influence the magnitude of response to dietary interventions. However, this factor could not be formally explored due to limited and inconsistently reported data across trials, including the lack of subgroup-specific outcome reporting across overlapping blood pressure ranges, which precluded stratified analyses according to clinically defined blood pressure categories. Third, the inclusion and pooling of both parallel and cross-over randomized controlled trials required methodological decisions that may have influenced the results. In particular, the combination of different study designs involves assumptions regarding the comparability of effect estimates, which may introduce additional uncertainty. Fourth, for cross-over trials, treatment effects were derived using within-participant comparisons following Cochrane Handbook recommendations. However, the included cross-over trials (n = 3) did not consistently report the necessary data to directly estimate paired standard errors, such as within-participant standard deviations or period-specific summary statistics. Therefore, standard errors were estimated assuming a within-participant correlation coefficient. Although a plausible value (r = 0.50) [17] was used and sensitivity analyses were conducted, the true correlation remains unknown and may have influenced the precision of the estimates. In addition, different statistical approaches were used to estimate pooled effects, including random-effects models with REML using both conventional and HKSJ adjustments. In some subgroup analyses, the use of HKSJ influenced the statistical significance of the results; however, overall findings remained consistent across approaches. Fifth, the relatively small sample sizes of the included trials may have contributed to imprecision in the effect estimates. Sixth, subgroup analyses by intervention duration were based on a limited number of studies, which may have reduced statistical power and contributed to imprecision, particularly for longer-term effects, and the potential overlap between intervention duration and study design (parallel vs. cross-over) may have influenced subgroup findings. Finally, a formal assessment of publication bias was not performed due to the limited number of studies available for most outcomes. Gray literature was not systematically searched, which may have led to the omission of relevant unpublished or non-indexed studies.

From a clinical perspective, the current evidence does not provide consistent evidence of an effect of berry supplementation on blood pressure in individuals with elevated blood pressure or hypertension, and remains limited and inconclusive for other vascular and cardiometabolic outcomes. Given the low to very low certainty of the evidence, these findings should be interpreted with caution.

From a research perspective, future randomized controlled trials with larger sample sizes and adequate statistical power are needed to better characterize the potential effects of berry supplementation on blood pressure and related cardiometabolic outcomes. In particular, future studies should aim to standardize intervention characteristics, including the type, dose, and duration of berry supplementation, and to report sufficient data to allow appropriate synthesis of cross-over and parallel designs. Future research should include more diverse populations, as most of the available evidence has been generated in high-income settings. Differences in dietary patterns, availability, and affordability of berries across settings may influence the feasibility and adherence to such interventions, potentially affecting their real-world applicability [38]. Moreover, additional research is needed to explore the effects of berry supplementation on 24 h ambulatory blood pressure and other vascular and cardiometabolic outcomes, as current evidence in these areas remains limited.

## 5. Conclusions

In conclusion, the current evidence does not support a consistent effect of berry supplementation on blood pressure and remains limited and inconclusive for other vascular and cardiometabolic outcomes. The overall certainty of the evidence is low to very low, precluding firm conclusions. Larger and methodologically rigorous randomized controlled trials are required to determine whether berry supplementation has any measurable effect on these outcomes.

## Figures and Tables

**Table 1:** Characteristics of included studies.

Author, Year	RCT Design	Country	Age (Years)	Clinical Population Characteristic	Sex	Sample Size	Intervention	Control	Duration	Funding
Loo, 2016 [28]	Cross-over	Finland	Range: 40–69	Elevated blood pressure (SBP 130–159 mmHg or DBP 85–99 mmHg)	Male: 14 Female: 24	n = 38	Active compound: Chokeberry juice + oven-dried chokeberry powder. Dose: 300 mL/day chokeberry juice + 3 g/day powder (18 g fresh chokeberries). Juice and powder mixed.	Active compound: Placebo juice + powder. Dose: 300 mL/day placebo juice + 3 g/day placebo powder. Juice and powder mixed	Intervention phase: 8 weeks. Control phase: 8 weeks. Wash out: No wash out	Tekes—the Finnish Funding Agency for Innovation
Richter, 2021 [31]	Cross-over	USA	Range: 30–65 Mean (SD): 47 (12)	Adults with elevated blood pressure (SBP 120–159 mmHg and/or DBP 80–99 mmHg)	Male: 25 Female: 15	n = 47	Active compound: 500 mL/day cranberry juice standardized to 27% juice (~70 kcal per serving). Dose: Two bottles per day (total 500 mL), either consumed at once or divided throughout the day	Active compound: 500 mL/day placebo juice, matched for color, calorie, and carbohydrate content. Dose: Two bottles per day (total 500 mL), either consumed at once or divided throughout the day	Intervention phase: 8 weeks. Control phase: 8 weeks (periods could extend to 12 weeks). Wash out: 8 weeks	Ocean Spray Cranberries, Inc. provided the juice
Keane, 2016 [26]	Cross-over	UK	Mean (SD): 31 (9)	Men with early hypertension (SBP ≥ 130 mmHg or DBP ≥ 80 mmHg)	Only male	n = 15	Active compound: Montmorency tart cherry juice. Dose: Single dose of 60 mL Montmorency tart cherry concentrate diluted with 100 mL water	Active compound: Placebo juice (low-fruit cordial [<1% fruit] diluted with water, whey protein isolate, maltodextrin). Dose: Single dose of 60 mL	Intervention phase: 8 h. Control phase: 8 h Wash out: ≥14 days	Cherry Marketing Institute and Northumbria University
Tjelle, 2015 [33]	Parallel	Norway	Range: 50–70	Adults with high normal BP (SBP 130–139 mmHg or DBP 85–89 mmHg) or stage 1–2 hypertension (SBP 140–179 mmHg or DBP 90–109 mmHg)	Male/Female Randomization number NR	n = 153 I. Dose 1: 51 I. Dose 2: 52 Control: 50	Active compound: 500 mL/day fruit-based beverages (MANA Blue [chokeberry and bilberry] and Optijuice [85% MANA Blue]). Dose: 500 mL/day.	Active compound: 500 mL/day placebo beverage. Dose: 500 mL/day	12 weeks	TINE SA and The Research Council of Norway
Woolf, 2023 [34]	Parallel	USA	Range: 40–65	Postmenopausal women with elevated BP or stage 1 hypertension (SBP 120–139 mmHg and/or DBP 80–89 mmHg)	Only female	n = 48 I: 24 C: 24	Active compound: 22 g/day freeze-dried blueberry powder. Dose: 22 g/day divided into 2 doses (11 g each). Morning and evening (approx. 6–8 h apart)	Active compound: 22 g/day placebo powder. Dose: 22 g/day divided into 2 doses (11 g each). Morning and evening (approx. 6–8 h apart)	12 weeks	U.S. Highbush Blueberry Council and NIH
Jeong, 2015 [22]	Parallel	South Korea	Mean (SD) Moderate dose: 60.2 (11.2) High dose: 55.5 (12.3) Control: 55.9 (12.8)	Prehypertensive patients not taking antihypertension medication (SBP 130–139 mm Hg or DBP 85–89 mm Hg)	Male: 24 Female: 21	n = 45 I. High dose: 15 I. Moderate dose: 15 C: 15	Active compound: Capsules of dried ripe black raspberries. Dose: Moderate dose: 1500 mg/day (8 capsules/day; 4 capsules twice daily). High dose: 2500 mg/day (4 capsules twice daily)	Active compound: Placebo capsules, identical in appearance to intervention. Dose: 8 capsules/day. Twice daily	8 weeks	Gochang Black Raspberry Research Institute
Kianbakhta, 2019 [27]	Parallel	Iran	Mean (SD) Intervention: 57 (6) Control: 61 (8)	Obese hypertensive outpatients (SBP 140–160 mmHg, DBP 80–100 mmHg)	Male/Female Randomization number NR	n = 112 I: 57 C: 55	Active compound: Capsules of 400 mg berry extract (V. arctostaphylos) powder. Dose: One capsule daily (3 times a day) alongside the standard anti-hypertensive treatment	Active compound: Placebo capsules (toast powder). Dose: One capsule daily (3 times a day) alongside the standard anti-hypertensive treatment	3 months	Research Institute for Islamic and Complementary Medicine
Johnson, 2015 /Johnson, 2017 [23,24]	Parallel	USA	Range: 45–65 Mean (SD) Intervention: 59.7 (4.6) Control: 57.3 (4.8)	Postmenopausal women with pre- or stage 1 hypertension (SBP 125–160 mmHg or DBP 85–90 mmHg)	Only female	n = 48 I: 25 C: 23	Active compound: Freeze-dried blueberry powder. Dose: 22 g freeze-dried blueberry powder split into 2 doses (11 g each), dissolved in water. Morning and evening with at least 6 to 8 h apart	Active compound: 22 g macronutrient-matched control powder. Dose: 22 g split into 2 doses (11 g each), dissolved in water. Morning and evening with at least 6 to 8 h apart	8 weeks	US Highbush Blueberry Council/US Department of Agriculture
Le Sayec, 2022 [32]	Parallel	UK	Mean (SD): 56.2 (8.7)	Prehypertensive adults (SBP 120–139 mmHg and/or DBP 80–89 mmHg)	Male: 47 Female: 55	n = 102 I: 51 C: 51	Active compound: Aronia berry extract capsules (Aronox^®^), 500 mg each. Dose: 1 capsule (500 mg)	Active compound: Identical capsules in appearance to the active capsules. Dose: 1 capsule (500 mg)	12 weeks	Grant from Naturex SA

I: Intervention; C: Control; SBP: Systolic blood pressure; DBP: Diastolic blood pressure; NR: Not reported; SD: Standard deviation; RCT: Randomized controlled trial.

**Table 6:** GRADE certainty of evidence of main outcomes.

Outcomes	Anticipated Absolute Effects * (95% CI)		№ of Participants (Studies)	Certainty of the Evidence (GRADE)	Comments
	Risk with Placebo	Risk with Berry			
Systolic blood pressure (mmHg) (SBP)	The mean systolic blood pressure (mmHg) was 0 mmHg	MD 2.35 mmHg lower (4.98 lower to 0.29 higher)	547 (9 RCTs) [22,23,26,27,28,31,32,33,34]	⨁◯◯◯ Very low ^a,b,c,d^	The evidence is very uncertain about the effect of Berry on systolic blood pressure (mmHg).
Diastolic blood pressure (mmHg) (DBP)	The mean diastolic blood pressure (mmHg) was 0 mmHg	MD 1.04 mmHg lower (2.83 lower to 0.76 higher)	547 (9 RCTs) [22,23,26,27,28,31,32,33,34]	⨁◯◯◯ Very low ^a,d,e,f^	The evidence is very uncertain about the effect of Berry on diastolic blood pressure (mmHg).
24 h-Systolic blood pressure (mmHg) (24 h-SBP) follow-up: range 8 weeks to 12 weeks	The mean 24 h- Systolic blood pressure (mmHg) was 0 mmHg	MD 0.90 mmHg lower (2.14 lower to 0.34 higher)	219 (4 RCTs) [22,28,31,32]	⨁⨁◯◯ Low ^g,h,i,j^	Berry may result in little to no difference in 24 h- Systolic blood pressure (mmHg).
24 h-Diastolic blood pressure (mmHg) (24 h-DBP) follow-up: range 8 weeks to 12 weeks	The mean 24 h- Diastolic blood pressure (mmHg) was 0 mmHg	MD 1.11 mmHg lower (2.04 lower to 0.17 lower)	219 (4 RCTs) [22,28,31,32]	⨁⨁◯◯ Low ^g,j,k,l^	Berry may not reduce 24 h-Diastolic blood pressure (mmHg).

* The risk in the intervention group (and its 95% confidence interval) is based on the assumed risk in the comparison group and the relative effect of the intervention (and its 95% CI).; CI: confidence interval; MD: mean difference; GRADE Working Group grades of evidence; High certainty: we are very confident that the true effect lies close to that of the estimate of the effect.; Moderate certainty: we are moderately confident in the effect estimate: the true effect is likely to be close to the estimate of the effect, but there is a possibility that it is substantially different.; Low certainty: our confidence in the effect estimate is limited; the true effect may be substantially different from the estimate of the effect. Very low certainty: we have very little confidence in the effect estimate; the true effect is likely to be substantially different from the estimated effect. Certainty of evidence is expressed using GRADE symbols: ⨁ indicates a filled rating and ◯ indicates an empty rating; two low (⨁⨁◯◯), and one very low certainty (⨁◯◯◯). Explanations: ^a^. Most of the meta-analysis weights correspond to trials whose overall risk of bias was judged as “low” or “unclear”; ^b^. Due to substantial heterogeneity (*I*^2^ = 65%, *p* value [*p* = 0.003]). The magnitude of the effect estimates varied considerably; ^c^. Due to serious imprecision. The confidence interval crossed one imprecision threshold (defined as 0.2 × SD. This corresponds to 4 mmHg); ^d^. Five out of the nine studies included in the meta-analysis were fully or partially funded by private organizations or industries related to the berry market; ^e^. Due to moderate heterogeneity (*I*^2^ = 56.6%, *p* value [*p* = 0.018]). Although the direction of effects was consistent across studies, the magnitude varied considerably, likely due to clinical and methodological diversity; ^f^. Not serious imprecision. Confidence interval does not cross imprecision threshold (defined as 0.2 × SD. This corresponds to 3 mmHg); ^g^. Most of the meta-analysis weights correspond to trials whose overall risk of bias was judged as “low” or “unclear”. However, point estimates did not substantially differ between “low” and “unclear” risk of bias studies; ^h^. Not important heterogeneity (*I*^2^ = 0%, *p* value [*p* = 0.891]). Effect estimates with overlapping confidence intervals. No evidence of opposing effects; ^i^. Serious imprecision. Confidence interval does not cross imprecision threshold (defined as 0.2 × SD. This corresponds to 3 mmHg) but the optimal information size (OIS, defined as n = 400 following GRADE guidelines) was not met; ^j^. Two out of the four studies included in the meta-analysis were fully or partially funded by private organizations or industries related to the berry market; ^k^. Not important heterogeneity (*I*^2^ = 0%, *p* value [*p* = 0.645]). Effect estimates with overlapping confidence intervals. No evidence of opposing effects; ^l^. Due to serious imprecision. The confidence interval crossed one imprecision threshold (defined as 0.2 × SD. This corresponds to 2 mmHg). The optimal information size (OIS, defined as n = 400 following GRADE guidelines) was not met.

## Data Availability

No new data were created or analyzed in this study. Data sharing is not applicable to this article.

## Supplementary Materials

The following supporting information can be downloaded at: https://www.mdpi.com/article/10.3390/nu18101504/s1, Supplementary Material S1: Search strategies; Supplementary Material S2: Detailed methods for data synthesis and statistical analysis; Supplementary Material S3: Full-text studies excluded after eligibility assessment and reasons for exclusion; Supplementary Table S1: Vascular outcomes; Supplementary Table S2. Lipid profile outcomes; Supplementary Table S3. Inflammatory outcomes; Figure S1: Augmentation index standardized at 75 beats per minute (%) forest plot; Figure S2: Flow-mediated dilation (%) forest plot; Figure S3: Pulse wave velocity: carotid–femoral pulse wave velocity (m/s) forest plot; Figure S4: Pulse wave velocity: brachial–aortic pulse wave velocity (m/s) forest plot; Figure S5: Total cholesterol (mg/dL) forest plot; Figure S6: HDL cholesterol (mg/dL) forest plot; Figure S7: LDL cholesterol (mg/dL) forest plot; Figure S8: Triglycerides (mg/dL) forest plot; Figure S9: C-reactive protein (mg/dL) forest plot; Figure S10: Tumor necrosis factor alpha (TNF-α) (pg/mL) forest plot; Figure S11: Systolic blood pressure (mmHg) sensitivity analysis (r = 0.25) forest plot; Figure S12: Systolic blood pressure (mmHg) sensitivity analysis (r = 0.75) forest plot; Figure S13: Diastolic blood pressure (mmHg) sensitivity analysis (r = 0.25) forest plot; Figure S14: Diastolic blood pressure (mmHg) sensitivity analysis (r = 0.75) forest plot; Figure S15: 24 h systolic blood pressure (mmHg) sensitivity analysis (r = 0.25) forest plot; Figure S16: 24 h systolic blood pressure (mmHg) sensitivity analysis (r = 0.75) forest plot; Figure S17: 24 h diastolic blood pressure (mmHg) sensitivity analysis (r = 0.25) forest plot; Figure S18: 24 h diastolic blood pressure (mmHg) sensitivity analysis (r = 0.75) forest plot; Figure S19: Augmentation Index at 75 bpm (%) sensitivity analysis (r = 0.25) forest plot; Figure S20: Augmentation Index at 75 bpm (%) sensitivity analysis (r = 0.75) forest plot; Figure S21: Carotid-femoral pulse wave velocity (m/s) sensitivity analysis (r = 0.25) forest plot; Figure S22: Carotid-femoral pulse wave velocity (m/s) sensitivity analysis (r = 0.75) forest plot; Figure S23: Total cholesterol (mg/dL) sensitivity analysis (r = 0.25) forest plot; Figure S24: Total cholesterol (mg/dL) sensitivity analysis (r = 0.75) forest plot; Figure S25: HDL cholesterol (mg/dL) sensitivity analysis (r = 0.25) forest plot; Figure S26: HDL cholesterol (mg/dL) sensitivity analysis (r = 0.75) forest plot; Figure S27: LDL cholesterol (mg/dL) sensitivity analysis (r = 0.25) forest plot; Figure S28: LDL cholesterol (mg/dL) sensitivity analysis (r = 0.75) forest plot; Figure S29: Triglycerides (mg/dL) sensitivity analysis (r = 0.25) forest plot; Figure S30: Triglycerides (mg/dL) sensitivity analysis (r = 0.75) forest plot; Figure S31: C-reactive protein (mg/dL) sensitivity analysis (r = 0.25) forest plot; Figure S32: C-reactive protein (mg/dL) sensitivity analysis (r = 0.75) forest plot; Supplementary Table S4: GRADE certainty of evidence of additional outcomes; Supplementary Table S5: GRADE evidence profile of main outcomes (resting systolic and diastolic blood pressure, and 24 h systolic and diastolic blood pressure); Supplementary Table S6: GRADE evidence profile of additional outcomes (vascular function markers, lipid profile, and inflammatory markers); Table S7: PRISMA 2020 Checklist.

## Abbreviations

The following abbreviations are used in this manuscript:

AIx75	Augmentation Index at 75 beats per minute
baPWV	Brachial–Aortic Pulse Wave Velocity
BP	Blood Pressure
CENTRAL	Cochrane Central Register of Controlled Trials
cfPWV	Carotid–Femoral Pulse Wave Velocity
CI	Confidence Interval
CRP	C-Reactive Protein
DBP	Diastolic Blood Pressure
DASH	Dietary Approaches to Stop Hypertension
FMD	Flow-Mediated Dilation
GRADE	Grading of Recommendations Assessment, Development and Evaluation
HDL	High-Density Lipoprotein
HKSJ	Hartung–Knapp–Sidik–Jonkman method
*I*^2^	Inconsistency Statistic
LDL	Low-Density Lipoprotein
MD	Mean Difference
MCP-1	Monocyte Chemoattractant Protein-1
MeSH	Medical Subject Headings
OIS	Optimal Information Size
PRISMA	Preferred Reporting Items for Systematic Reviews and Meta-Analyses
PROSPERO	International Prospective Register of Systematic Reviews
RCT	Randomized Controlled Trial
REML	Restricted Maximum Likelihood
RoB	Risk of Bias
SBP	Systolic Blood Pressure
SoF	Summary of Findings
TNF-α	Tumor Necrosis Factor alpha
SD	Standard Deviation
SE	Standard Error
