# Supplementary material for: Effects of Oral Berry Supplementation on Blood Pressure in Adults with Hypertension or Elevated Blood Pressure: A Systematic Review and Meta-Analysis of Randomized Controlled Trials
Source: Nutrients. 2026 May 8;18(10):1504. doi: 10.3390/nu18101504 (PMC13209865; doi:10.3390/nu18101504)
Supplement: Supplementary file 1 [file nutrients-18-01504-s001.zip › nutrients-4275927-supplementary.pdf]

Supplementary Material

Effects of oral berry supplementation on blood pressure in adults with hypertension or elevated blood pressure: A systematic review and meta-analysis of Randomized controlled trials

Table of Contents

Supplementary material 1. Search strategies…………………………………...…..3

Supplementary material 2. Detailed methods for data synthesis and statistical analysis ……………………………………………………………………….………7

Supplementary material 3. Full-text studies excluded after eligibility assessment and reasons for exclusion …..…………………………………………………………..12

Supplementary Table S1……………………………………………………………13

Supplementary Table S2……………………………………………………………15

Supplementary Table S3……………………………………………………………19

Supplementary Figure 1……………………………………………………………20

Supplementary Figure 2……………………………………………………………20

Supplementary Figure 3……………………………………………………………20

Supplementary Figure 4……………………………………………………………21

Supplementary Figure 5……………………………………………………………21

Supplementary Figure 6……………………………………………………………22

Supplementary Figure 7……………………………………………………………22

Supplementary Figure 8……………………………………………………………23

Supplementary Figure 9……………………………………………………………23

Supplementary Figure 10..…………………………………………………………24

Supplementary Figure 11..…………………………………………………………25

Supplementary Figure 12..…………………………………………………………25

Supplementary Figure 13..…………………………………………………………26

Supplementary Figure 14..…………………………………………………………26

Supplementary Figure 15..........27

Supplementary Figure 16..........27

Supplementary Figure 17..........28

Supplementary Figure 18..........28

Supplementary Figure 19..........29

Supplementary Figure 20..........29

Supplementary Figure 21..........30

Supplementary Figure 22..........30

Supplementary Figure 23..........31

Supplementary Figure 24..........31

Supplementary Figure 25..........32

Supplementary Figure 26..........32

Supplementary Figure 27..........33

Supplementary Figure 28..........33

Supplementary Figure 29..........34

Supplementary Figure 30..........34

Supplementary Figure 31..........35

Supplementary Figure 32..........35

Supplementary Table S4..........36

Supplementary Table S5..........41

Supplementary Table S6..........43

Supplementary Table S7..........47

Supplementary material 1. Search strategies.

**Database: PubMed<br>Date of the search: May 12, 2025**		
**Search**	**Terms**	**Results**
#1	Hypertension[Mesh]	331,687
#2	Hypertens*[tiab]	560,920
#3	Blood Pressure*[tiab]	376,119
#4	High Blood[tiab]	27,312
#5	Prehypertension[Mesh]	1,233
#6	Prehypertension*[tiab]	2,644
#7	Pre-Hypertension*[tiab]	627
#8	Antihypertensive*[tiab]	65,098
#9	#1 OR #2 OR #3 OR #4 OR #5 OR #6 OR #7 OR #8	860,270
#10	Blueberry Plants[Mesh]	1,704
#11	Vaccinium[tiab]	2,420
#12	Berry[tiab]	8,428
#13	Berries[tiab]	6,244
#14	Blueberr*[tiab]	3,929
#15	Strawber*[tiab]	7,534
#16	Blackberr*[tiab]	1,241
#17	Whiteberr*[tiab]	21
#18	Cranberr*[tiab]	2,240
#19	Cherry[tiab]	6,475
#20	Cherries[tiab]	1,162
#21	Bilberr*[tiab]	774
#22	Raspberr*[tiab]	3,504
#23	Tart Cherr*[tiab]	221
#24	Chokeberr*[tiab]	511
#25	#10 OR #11 OR #12 OR #13 OR #14 OR #15 OR #16 OR #17 OR #18 OR #19 OR #20 OR #21 OR #22 OR #23 OR #24	35,776
#26	#9 AND #25	585
#27	((Randomized Controlled Trial[pt] OR Controlled Clinical Trial[pt] OR Randomized Controlled Trials[Mesh] OR Random Allocation[Mesh] OR Double-Blind Method[Mesh] OR Single-Blind Method[Mesh] OR Clinical Trial[pt] OR Clinical Trials[Mesh]) OR (Clinical Trial[tw]) OR ((Singl*[tw] OR Doubl*[tw] OR Trebl*[tw] OR Tripl*[tw]) AND (Mask*[tw] OR Blind*[tw])) OR (Placebos[Mesh] OR Placebo*[tw] OR Random*[tw] OR Research Design [mh:noexp]) NOT (Animals [Mesh] NOT Human[Mesh]))	2,399,512
#28	#26 AND #27	202

**Database: CENTRAL (Cochrane Central Register of Controlled Trials)<br>Date of the search: May 12, 2025**

Search	Terms	Results
#1	MH Blueberry Plants	1
#2	Vaccinium:ti,ab,kw	305
#3	Berr*:ti,ab,kw	889
#4	Blueberr*:ti,ab,kw	358
#5	Strawber*:ti,ab,kw	385
#6	Blackberr*:ti,ab,kw	65
#7	Whiteberr*:ti,ab,kw	0
#8	Cranberr*:ti,ab,kw	540
#9	Bilberr*:ti,ab,kw	144
#10	Cherry:ti,ab,kw	420
#11	Cherries:ti,ab,kw	95
#12	Bilberr*:ti,ab,kw	144
#13	Raspberr*:ti,ab,kw	154
#14	Tart Cherr*:ti,ab,kw	144
#15	Chokeberr*:ti,ab,kw	37
#16	#1 OR #2 OR #3 OR #4 OR #5 OR #6 OR #7 OR #8 OR #9 OR #10 OR #11 OR #12 OR #13 OR #14 OR #15	2725
#17	MH Hypertension	1574
#18	Hypertens*:ti,ab,kw	81599
#19	(Blood NEAR/1 Pressure*):ti,ab,kw	122150
#20	(High NEAR/1 Blood):ti,ab,kw	4416
#21	MH Prehypertension	18
#22	Prehypertension*:ti,ab,kw	786
#23	Pre-Hypertension*:ti,ab,kw	205
#24	Antihypertensive*:ti,ab,kw	23239
#25	#17 OR #18 OR #19 OR #20 OR #21 OR #22 OR #23 OR #24	168619
#26	#16 AND #25 in Trials	410

**Database: Embase (Ovid)**<br>**Date of the search: May 12, 2025**		
**Search**	**Terms**	**Results**
#1	exp hypertension/	1108135
#2	Hypertens*.ti,ab.	859540
#3	(Blood adj1 Pressure*).ti,ab.	539379
#4	(High adj1 Blood).ti,ab.	41680
#5	exp prehypertension/	4102
#6	Prehypertension*.ti,ab.	3032
#7	Pre-Hypertension*.ti,ab.	1208
#8	Antihypertensive*.ti,ab.	88124
#9	or/1-8	1666499
#10	exp Vaccinium/	6709
#11	Vaccinium.ti,ab.	2296
#12	Berry.ti,ab.	7755

#13	Berries.ti,ab.	6530
#14	Blueberr*.ti,ab.	3999
#15	Strawber*.ti,ab.	7446
#16	Blackberr*.ti,ab.	1271
#17	Whiteberr*.ti,ab.	0
#18	Cranberr.ti,ab.	0
#19	Bilberr*.ti,ab.	843
#20	Cherry.ti,ab.	6833
#21	Cherries.ti,ab.	1141
#22	Bilberr*.ti,ab.	843
#23	Raspberr*.ti,ab.	3663
#24	Tart-Cherr*.ti,ab.	254
#25	Chokeberr*.ti,ab.	456
#26	or/10-25	37081
#27	9 and 26	1264
#28	((Random$ or Placebo$ or ((Single$ or Double$ or Triple$ or Treble$) and (Blind$ or Mask$)) or Controlled Clinical Trial$).ti,ab. or Retracted Article/) not (animal$ not human$).sh,hw.	2253153
#29	27 and 28	311

**Database: Scopus<br>Date of the search: May 12, 2025**		
**Search**	**Terms**	**Results**
#1	(INDEXTERMS (hypertension) OR TITLE-ABS (hypertens*) OR TITLE-ABS ("Blood Pressure*") OR TITLE-ABS ("High Blood") OR INDEXTERMS (prehypertension) OR TITLE-ABS (prehypertension*) OR TITLE-ABS (pre-hypertension*) OR TITLE-ABS (antihypertensive*)) AND (INDEXTERMS ("Blueberry Plants") OR TITLE-ABS (vaccinium) OR TITLE-ABS (berry) OR TITLE-ABS (berries) OR TITLE-ABS (blueberr*) OR TITLE-ABS (strawber*) OR TITLE-ABS (blackberr*) OR TITLE-ABS (whiteberr*) OR TITLE-ABS (cranberr*) OR TITLE-ABS (bilberr*) OR TITLE-ABS (cherry) OR TITLE-ABS (cherries) OR TITLE-ABS (bilberr*) OR TITLE-ABS (raspberr*) OR TITLE-ABS ("Tart Cherr*") OR TITLE-ABS (chokeberr*)) AND (INDEXTERMS ("clinical trials" OR "clinical trials as a topic" OR "randomized controlled trial" OR "Randomized Controlled Trials as Topic" OR "controlled clinical trial" OR "Controlled Clinical Trials" OR "random allocation" OR "Double-Blind Method" OR "Single-Blind Method" OR "Cross-Over Studies" OR "Placebos" OR "multicenter study" OR "double blind procedure" OR "single blind procedure" OR "crossover procedure" OR "clinical trial" OR "controlled study" OR "randomization" OR "placebo")) OR (TITLE-ABS-KEY (("clinical trials" OR "clinical trials as a topic"	378

	OR "randomized controlled trial" OR "Randomized Controlled Trials as Topic" OR "controlled clinical trial" OR "Controlled Clinical Trials as Topic" OR "random allocation" OR "randomly allocated" OR "allocated randomly" OR "Double-Blind Method" OR "Single-Blind Method" OR "Cross-Over Studies" OR "Placebos" OR "cross-over trial" OR "single blind" OR "double blind" OR "factorial design" OR "factorial trial"))) OR (TITLE-ABS (clinical AND trial* OR trial* OR rct* OR random* OR blind*))	

Supplementary material 2. Detailed methods for data synthesis and statistical analysis

Meta-analyses combined evidence from randomized controlled trials (RCTs) with parallel and cross-over designs. For all outcomes, mean differences (MDs) and corresponding standard errors (SEs) were extracted directly when reported or derived following recommendations from the Cochrane Handbook (Chapter 23).

For cross-over trials, paired MDs were estimated using within-participant comparisons. When paired SEs were not reported, they were approximated using the mean, confidence interval and a t-distribution (n-1 degrees of freedom) or the standard deviation of within-participant differences (SD$diff$), as appropriate. For parallel-group trials, MDs and SEs were calculated using standard formulas for independent groups. When trials included multiple intervention arms sharing a single control group, intervention arms were combined prior to analysis to avoid unit-of-analysis errors, following Cochrane Handbook guidance (Chapter 6, Section 6.5.2.10; Chapter 23, Section 23.3).

All effect estimates were pooled using the generic inverse-variance method.

Main outcomes

Resting systolic and diastolic blood pressure

Meta-analyses were conducted combining both parallel (n = 6) and cross-over (n = 3) RCT designs.

For cross-over trials, MDs were calculated as the difference between intervention and control end-period means.

In the trial by Loo et al. (2016), paired SEs were not reported; therefore, the SE was derived from the reported confidence interval (CI) assuming a two-sided t-distribution with 36 degrees of freedom (n = 37 participants) (t = 2.028), calculated as:

$$SE = \frac{MD - lower\ CI}{t}$$

$(Equation\ 1)$

In the cross-over trials by Keane et al. (2016) and Richter et al. (2021), SEs were derived using the SD of within-participant differences (SD_{diff}), calculated as:

$$SD_{diff} = \sqrt{SD_{intervention}^2 + SD_{control}^2 - (2 \times Corr \times SD_{intervention} \times SD_{control})}$$

$(Equation\ 2)$

A within-participant correlation coefficient (r) of 0.5 was assumed for the main analysis; sensitivity analyses were conducted using r = 0.25 and r = 0.75. SEs were then calculated as:

$$SE = \frac{SD_{diff}}{\sqrt{N}}$$

$(Equation\ 3)$

For parallel-group trials, MDs were calculated as the difference between post-intervention means. Corresponding SEs were calculated using group-specific standard deviations and sample sizes, according to standard formulas for independent groups. The following equation was used:

$$SE = \sqrt{\frac{SD_{intervention}^2}{n_{intervention}} + \frac{SD_{control}^2}{n_{control}}}$$

$(Equation\ 4)$

In the parallel-group trials by Tjelle et al. (2015) and Jeong et al. (2015), which included two intervention arms and a single control group, the intervention arms were combined into a single group to avoid unit-of-analysis errors, following Cochrane Handbook recommendations (Chapter 6, Section 6.5.2.10; Chapter 23, Section 23.3). Combined intervention means and standard deviations were calculated prior to estimating the MD and corresponding SE for SBP and DBP using the post-combination intervention group data. The following equations were used for combining groups:

$$Sample\ size = N_1 + N_2$$

$$Mean = \frac{N_1 Mean_1 + N_2 Mean_2}{N_1 + N_2}$$

$$SD = \sqrt{\frac{(N_1 - 1)SD_1^2 + (N_2 - 1)SD_2^2 + \frac{N_1 N_2}{N_1 + N_2}(Mean_1^2 + Mean_2^2 - 2Mean_1 Mean_2)}{N_1 + N_2 - 1}}$$

$(Equation\ 5)$

All MDs and corresponding SEs from cross-over and parallel-group trials were combined using the generic inverse-variance method.

24-hour systolic and diastolic blood pressure

Meta-analyses were conducted combining both parallel (n = 2) and cross-over (n = 2) RCT, following the same analytical principles described for resting blood pressure.

In the cross-over trial by Loo et al. (2016), the MD and corresponding confidence CI were reported. The SE was calculated using Equation 1, and assuming a two-sided t-distribution with 36 degrees of freedom (n = 37 participants) (t = 2.028)

In the cross-over trial by Richter et al. (2021), the MD was calculated as the difference between intervention and control period means. The SE was approximated using the SD of within-participant differences with a correlation coefficient (r) of 0.5 for the main analysis and r = 0.25 and r = 0.75 for sensitivity analyses (Equation 2 and Equation 3)

In parallel-group trials, MDs and corresponding SEs were calculated as described for resting systolic and diastolic blood pressure. In the trial by Jeong et al. (2015), which included two intervention arms and a single control group, the intervention arms were first combined into a single group following Cochrane Handbook recommendations to avoid unit-of-analysis errors (Equation 5); MDs and SEs were then calculated using the post-combination group-level data (Equation 4).

All MDs and corresponding SEs from cross-over and parallel-group trials were combined using the generic inverse-variance method.

Additional outcomes

Pulse wave velocity (pwv)

Carotid–femoral and brachial–aortic PWV were analyzed separately. For carotid–femoral PWV, parallel (n = 2) and cross-over (n = 2) RCTs were combined; for brachial–aortic PWV, only parallel trials (n = 2) were included.

Before any analysis all PWV values were converted to m/s (cm/s ÷ 100) when required as this is the measure unit used for pulse wave velocity (1). For Jeong et al. (2015), only right-arm measurements were used, following prior methodological recommendations (1). For Woolf et al. (2023), mean values and SEs at 12 weeks were extracted from figures using PlotDigitizer (https://plotdigitizer.sourceforge.net/).

In the cross-over trial by Richter et al. (2021), SEs were estimated using SDdiff (Equation 2) assuming r = 0.5 (sensitivity analyses: r = 0.25 and 0.75), followed by Equation 3. Parallel-group trials were analyzed using Equation 4, with intervention arms combined where applicable using Equation 5.

Augmentation index at 75 bpm (Aix75)

Parallel (n=2) and cross-over (n = 1) RCT were meta analyzed. For the cross-over trial (Richter et al., 2021), MDs and SEs were estimated using Equations 2 and 3, assuming r = 0.5 (sensitivity analyses: r = 0.25 and 0.75). Parallel-group trials were analyzed using Equation 4.

Flow mediated dilation (FMD)

Two parallel RCT were meta analyzed. MDs and SEs were calculated using Equation 4. For Woolf et al. (2023), baseline-to-12-week change values (mean, lower and upper SE) were extracted from figures using PlotDigitizer (https://plotdigitizer.sourceforge.net/).

Cholesterol outcomes (HDL-c, LDL-c, and total cholesterol)

Meta-analyses for HDL-c, LDL-c, and total cholesterol parallel and cross-over RCT designs, as available for each outcome (HLD-c: parallel [n=2] cross-over [n=2]; LDL-c: parallel [n=2] cross-over [n=1]; Total cholesterol: parallel [n=2] cross-over [n=2]).

To ensure consistency across studies, values reported in mmol/L were converted to mg/dL prior to analysis using a conversion factor of 0.0259, according to standard SI-to-conventional unit conversion guidelines (https://academic.oup.com/amamanualofstyle/si-conversion-calculator)

For parallel trials MD was obtained as difference between intervention and control period means and SE was estimated using Equation 4.

For cross-over trials, paired MDs were calculated as the difference between intervention and control period means. The approach used to derive paired SEs differed by outcome and study, depending on data availability, as detailed below.

- *HDL cholesterol (HDL-c)*: In Loo et al. (2016), paired SEs were derived from the reported mean differences and 95% CI, assuming a two-sided paired t-distribution with 36 degrees of freedom (n = 37 participants) (t = 2.028). In Richter et al. (2021), paired SEs were estimated using Equation 2 assuming a within-participant correlation coefficient of 0.5 for the main analysis, with sensitivity analyses conducted using correlation coefficients of 0.25 and 0.75.
- *LDL cholesterol (LDL-c):* In the cross-over trial by Richter et al. (2021), paired SEs were estimated using the SD$diff$ approach (Equation 2), assuming a within-participant correlation coefficient of 0.5 for the main analysis, with sensitivity analyses using coefficients of 0.25 and 0.75.
- *Total cholesterol:* In Loo et al. (2016), paired SEs were derived from reported CI assuming a two-sided paired t-distribution, with 36 degrees of freedom (n = 37 participants) (t = 2.028), In Richter et al. (2021), paired SEs were estimated using the SD$diff$ approach (Equation 2), assuming a within-participant correlation coefficient of 0.5 for the main analysis, with sensitivity analyses conducted using coefficients of 0.25 and 0.75.

Triglycerides

Meta-analyses for triglycerides included randomized controlled trials with parallel and cross-over designs (parallel [n = 2], cross-over [n = 2]).

To ensure consistency across studies, triglyceride values reported in mmol/L were converted to mg/dL prior to analysis using a conversion factor of 0.0113, according to

standard SI-to-conventional unit conversion guidelines (https://academic.oup.com/amamanualofstyle/si-conversion-calculator).

For parallel RCT, MDs were calculated as the difference between post-intervention means in the intervention and control groups and SE were estimated using Equation 4.

For cross-over trials, paired MDs were calculated as the difference between intervention and control period means. The SE in the Loo et al. (2016) trial, was calculated using the mean, and CI values and assuming a two-sided t-distribution with 36 degrees of freedom (n = 37 participants) (t = 2.028), whereas for Richtcher et al. (2021) the SE was approximated using Equation 2 (assuming correlation coefficient of 0.5 as main analysis and 0.25 and 0.75 as sensitivity) and Equation 3.

C reactive Protein

Parallel (n=2) and cross-over (n = 1) randomized controlled trial designs were meta analyzed. To ensure consistency across studies, CRP values reported in mg/L were converted to mg/dL prior to analysis using a conversion factor of 10, according to standard SI-to-conventional unit conversion guidelines (https://academic.oup.com/amamanualofstyle/si-conversion-calculator).

For Richtcher et al. (2021) the SE was approximated using Equation 2 (assuming correlation coefficient of 0.5 as main analysis and 0.25 and 0.75 as sensitivity) and Equation 3.

In the parallel-group trials by Johnson et al. (2015) and Jeong et al. (2015), which included two intervention arms and a single control group, the intervention arms were combined into a single group using Equation 5 for SE. Combined intervention means and standard deviations were calculated prior to estimating the MD and corresponding SE (using Equation 4)

TNF-alfa

Parallel (n=1) and cross-over (n = 1) RCT designs were meta analyzed. For the Loo et al. (2016) trial, the SE was calculated using the mean, CI values and assuming a two-sided t-distribution with 36 degrees of freedom (n = 37 participants) (t = 2.028). The MD for the parallel trial was calculated as difference between intervention and control period means and SE was estimated using Equation 4.

References

1. Spronck B, Terentes-Printzios D, Avolio AP, Boutouyrie P, Guala A, Jerončić A, et al. 2024 recommendations for validation of noninvasive arterial pulse wave velocity measurement devices. Hypertension. 2024 Jan;81(1):183-192. doi:10.1161/HYPERTENSIONAHA.123.21618

Supplementary Material 3. Full-text studies excluded after eligibility assessment and reasons for exclusion

Study	Reason for exclusion
Novaes-Gaeta, 2024	Non-randomized study design
McAnulty, 2019	Hypertension/elevated BP status not reported
Kardum, 2015	Non-randomized study design
Bhaswant, 2019	Intervention outside predefined berry list

References

Novaes-Gaeta, L.; Moraes, M.; Katayama, K.; Sangaletti, C.; Irigoyen, M.; Freitas, S.; Viana, A.; Caldini, E.; Ferreira Lopes, H. Effect of Antioxidant Capsule Supplementation on Oxidative Stress Markers in Hypertensive Patients. Scripta Medica 2024, 55, 685–695, doi:10.5937/scriptamed55-54200.

McAnulty, L.S.; Collier, S.R.; Pike, J.; Thompson, K.L.; McAnulty, S.R. Time course of blueberry ingestion on measures of arterial stiffness and blood pressure. Journal of Berry Research 2019, 9, 631–640, doi:10.3233/JBR-190413.

Kardum, N.; Milovanović, B.; Šavikin, K.; Zdunić, G.; Mutavdžin, S.; Gligorijević, T.; Spasić, S. Beneficial Effects of Polyphenol-Rich Chokeberry Juice Consumption on Blood Pressure Level and Lipid Status in Hypertensive Subjects. Journal of Medicinal Food 2015, 18, 1231–1238, doi:10.1089/jmf.2014.0171.

Bhaswant, M.; Brown, L.; Mathai, M.L. Queen Garnet plum juice and raspberry cordial in mildly hypertensive obese or overweight subjects: A randomized, double-blind study. Journal of Functional Foods 2019, 56, 119–126, doi:https://doi.org/10.1016/j.jff.2019.03.011.

Supplementary Table S1. Vascular outcomes.

Group / Subgroup	Analysis specification	Studies (k)	Participants (N)	RE Method	Mean Difference (95% CI)	p-value	I^2	Included studies
**Augmentation Index at 75 bpm (AIx@75)**								
Overall	Parallel RCT + Cross over RCT (r = 0.50)	3	180	REML + HKSJ	-0.58 (-6.35, 5.20)	0.710	42.5	Woolf, 2023; Le Sayec, 2022; Richter, 2021
				REML + classic	-0.58 (-2.93, 1.78)	0.632	42.5	
	Parallel RCT	2	140	REML + HKSJ	-1.31 (-34.97, 32.36)	0.708	67.0	Woolf, 2023; Le Sayec, 2022
				REML + classic	-1.31 (-6.50, 3.89)	0.622	67.0	
	Cross over RCT (r = 0.50)	1	40	REML + HKSJ	0.00 (-1.96, 1.96)	1.000	NA	Richter, 2021
				REML + classic	0.00 (-1.96, 1.96)	1.000	NA	
Subgroup: Duration	> 8 weeks	2	140	REML + HKSJ	-1.31 (-34.97, 32.36)	0.708	67.0	Woolf, 2023; Le Sayec, 2022
				REML + classic	-1.31 (-6.50, 3.89)	0.622	67.0	
	≤ 8 weeks*	1	40	REML + HKSJ	0.00 (-1.96, 1.96)	1.000	NA	Richter, 2021
				REML + classic	0.00 (-1.96, 1.96)	1.000	NA	
**Flow-mediated dilation (FMD)**								
Overall	Only Parallel RCT	2	140	REML + HKSJ	0.41 (-12.57, 13.39)	0.758	73.3	Woolf, 2023; Le Sayec, 2022
				REML + classic	0.41 (-1.59, 2.41)	0.689	73.3	
**Carotid-femoral pulse wave velocity (m/s)**								
Overall	Parallel RCT + Cross over RCT (r = 0.50)	4	220	REML + HKSJ	0.04 (-0.21, 0.28)	0.673	0.0	Woolf, 2023; Johnson 2015 & Johnson 2017; Le Sayec, 2022; Richter, 2021
				REML + classic	0.04 (-0.14, 0.21)	0.691	0.0	
	Parallel RCT	3	180	REML + HKSJ	0.22 (-0.58, 1.01)	0.366	0.0	Woolf, 2023; Johnson 2015 & Johnson 2017; Le Sayec, 2022
				REML + classic	0.22 (-0.22, 0.65)	0.332	0.0	
	Cross over RCT (r = 0.50)	1	40	REML + HKSJ	0.00 (-0.20, 0.20)	1.000	NA	Richter, 2021
				REML + classic	0.00 (-0.20, 0.20)	1.000	NA	
Subgroup: Duration	> 8 weeks	2	140	REML + HKSJ	0.25 (-3.11, 3.61)	0.522	21.6	Woolf, 2023; Le Sayec, 2022
				REML + classic	0.25 (-0.27, 0.76)	0.351	21.6	
	≤ 8 weeks*	2	80	REML + HKSJ	-0.00 (-0.04, 0.04)	0.910	0.0	Johnson 2015 & Johnson 2017; Richter, 2021
				REML + classic	-0.00 (-0.19, 0.19)	0.997	0.0	

Group / Subgroup	Analysis specification	Studies (k)	Participants (N)	RE Method	Mean Difference (95% CI)	p-value	I^2	Included studies
Subgroup: Supplement presentation	Freeze-dried powder	2	83	REML + HKSJ	0.42 (-2.35, 3.20)	0.304	0.0	Woolf, 2023; Johnson 2015 & Johnson 2017
				REML + classic	0.42 (-0.19, 1.03)	0.174	0.0	
	Capsule	1	97	REML + HKSJ	0.00 (-0.62, 0.62)	1.000	NA	Le Sayec, 2022
				REML + classic	0.00 (-0.62, 0.62)	1.000	NA	
	Juice*	1	40	REML + HKSJ	0.00 (-0.20, 0.20)	1.000	NA	Richter, 2021
				REML + classic	0.00 (-0.20, 0.20)	1.000	NA	
Subgroup: Berry type	Blueberry	2	83	REML + HKSJ	0.42 (-2.35, 3.20)	0.304	0.0	Woolf, 2023; Johnson 2015 & Johnson 2017
				REML + classic	0.42 (-0.19, 1.03)	0.174	0.0	
	Chokeberry	1	97	REML + HKSJ	0.00 (-0.62, 0.62)	1.000	NA	Le Sayec, 2022
				REML + classic	0.00 (-0.62, 0.62)	1.000	NA	
	Cranberry*	1	40	REML + HKSJ	0.00 (-0.20, 0.20)	1.000	NA	Richter, 2021
				REML + classic	0.00 (-0.20, 0.20)	1.000	NA	
**Brachial-aortic pulse wave velocity (m/s)**								
Overall	Only Parallel RCT	2	85	REML + HKSJ	-0.26 (-8.39, 7.87)	0.755	44.9	Johnson 2015 & Johnson 2017; Jeong, 2015
				REML + classic	-0.26 (-1.51, 0.99)	0.685	44.9	

REML: Restricted Maximum Likelihood; HKSJ: Hartung-Knapp-Sidik-Jonkman; NA: Not applicable

For parallel RCT with two intervention arms compilation in one single arm was performed as recommended by Cochrane manual (Chapter 6; 6.5.2.10) using sd: $\sqrt{[(n1-1)\cdot sd1^2 + (n2-1)\cdot sd2^2 + (n1\cdot n2/(n1+n2))\cdot(mean1^2 + mean2^2 - 2\cdot mean1\cdot mean2)] / (n1+n2-1)}$

Mean difference was calculated for cross-over RCT using correlation coefficients 0.5, 0.25 and 0.75 as recommended by Cochrane manual (Chapter 6; 6.5.2.10) using $(sd\_treat^2 + sd\_cont^2 - 2\cdot r\cdot sd\_treat\cdot sd\_cont)$

Parallel RCT with 3 arms: Tjelle 2015 and Jeong, 2015

*For subgroup analysis all cross-over RCT were included considering a correlation of 0.5 for paired differences

**For subgroup analysis only Richter, 2021 cross-over RCT was included considering a correlation of 0.5 for paired differences. For the rest of cross-over RCT the reported mean difference was used

Supplementary Table S2. Lipid profile outcomes.

Group / Subgroup	Analysis specification	Studies (k)	Participants (N)	RE Method	Mean Difference (95% CI)	p-value	I^2	Included studies
**Total Cholesterol (mg/dL)**								
Overall	Parallel RCT + Cross over RCT (r = 0.50)	4	217	REML + HKSJ	0.11 (-4.21, 4.43)	0.941	0.0	Loo, 2016; Richter, 2021; Woolf, 2023; Le Sayec, 2022
				REML + classic	0.11 (-4.58, 4.80)	0.963	0.0	
	Parallel RCT	2	140	REML + HKSJ	2.29 (-65.88, 70.45)	0.744	0.0	Woolf, 2023; Le Sayec, 2022
				REML + classic	2.29 (-9.64, 14.21)	0.707	0.0	
	Cross over RCT (r = 0.50)	2	77	REML + HKSJ	-0.29 (-5.39, 4.81)	0.912	0.0	Loo, 2016; Richter, 2021
				REML + classic	-0.29 (-6.65, 6.08)	0.668	0.0	
Subgroup: Duration	> 8 weeks	2	140	REML + HKSJ	2.29 (-65.88, 70.45)	0.744	0.0	Woolf, 2023; Le Sayec, 2022
				REML + classic	2.29 (-9.64, 14.21)	0.707	0.0	
	≤ 8 weeks*	2	77	REML + HKSJ	-0.29 (-6.65, 6.08)	0.668	0.0	Loo, 2016; Richter, 2021
				REML + classic	-0.29 (-5.39, 4.81)	0.912	0.0	
Subgroup: Supplement presentation	Freeze-dried powder	1	43	REML + HKSJ	-3.00 (-19.74, 13.74)	0.725	NA	Woolf, 2023
				REML + classic	-3.00 (-19.74, 13.74)	0.725	NA	
	Capsule	1	97	REML + HKSJ	7.73 (-9.26, 24.72)	0.373	NA	Le Sayec, 2022
				REML + classic	7.73 (-9.26, 24.72)	0.373	NA	
	Juice*	2	77	REML + HKSJ	-0.29 (-6.65, 6.08)	0.668	0.0	Loo, 2016; Richter, 2021
				REML + classic	-0.29 (-5.39, 4.81)	0.912	0.0	
Subgroup: Berry type	Blueberry	1	43	REML + HKSJ	7.73 (-9.26, 24.72)	0.373	NA	Woolf, 2023
				REML + classic	7.73 (-9.26, 24.72)	0.373	NA	
	Chokeberry*	2	134	REML + HKSJ	-0.29 (-6.65, 6.08)	0.668	0.0	Le Sayec, 2022; Loo, 2016
				REML + classic	-0.29 (-5.39, 4.81)	0.912	0.0	
	Cranberry*	1	40	REML + HKSJ	-3.00 (-19.74, 13.74)	0.725	NA	Richter, 2021
				REML + classic	-3.00 (-19.74, 13.74)	0.725	NA	
**HDL Cholesterol (mg/dL)**								
Overall	Parallel RCT + Cross over RCT (r = 0.50)	4	217	REML + HKSJ	-0.49 (-2.21, 1.23)	0.431	0.0	Loo, 2016; Richter, 2021; Woolf, 2023; Le Sayec, 2022
				REML + classic	-0.49 (-1.81, 0.83)	0.466	0.0	

Group / Subgroup	Analysis specification	Studies (k)	Participants (N)	RE Method	Mean Difference (95% CI)	p-value	I^2	Included studies
	Parallel RCT	2	140	REML + HKSJ	1.94 (-29.01, 32.89)	0.572	0.0	Woolf, 2023; Le Sayec, 2022
				REML + classic	1.94 (-4.21, 8.08)	0.537	0.0	
	Cross over RCT (r = 0.50)	2	77	REML + HKSJ	-0.61 (-7.97, 6.75)	0.484	0.0	Loo, 2016; Richter, 2021
				REML + classic	-0.61 (-1.96, 0.74)	0.377	0.0	
Subgroup: Duration	> 8 weeks	2	140	REML + HKSJ	1.94 (-29.01, 32.89)	0.572	0.0	Woolf, 2023; Le Sayec, 2022
				REML + classic	1.94 (-4.21, 8.08)	0.537	0.0	
	≤ 8 weeks**	2	77	REML + HKSJ	-0.61 (-7.97, 6.75)	0.484	0.0	Loo, 2016; Richter, 2021
				REML + classic	-0.61 (-1.96, 0.74)	0.377	0.0	
Subgroup: Supplement presentation	Freeze-dried powder	1	43	REML + HKSJ	5.00 (-4.87, 14.87)	0.321	NA	Woolf, 2023
				REML + classic	5.00 (-4.87, 14.87)	0.321	NA	
	Capsule	1	97	REML + HKSJ	0.00 (-7.85, 7.85)	1.000	NA	Le Sayec, 2022
				REML + classic	0.00 (-7.85, 7.85)	1.000	NA	
	Juice**	2	77	REML + HKSJ	-0.61 (-7.97, 6.75)	0.484	0.0	Loo, 2016; Richter, 2021
				REML + classic	-0.61 (-1.96, 0.74)	0.377	0.0	
Subgroup: Berry type	Blueberry	1	43	REML + HKSJ	5.00 (-4.87, 14.87)	0.321	NA	Woolf, 2023
				REML + classic	5.00 (-4.87, 14.87)	0.321	NA	
	Chokeberry	2	134	REML + HKSJ	-1.10 (-4.41, 2.22)	0.148	0.0	Le Sayec, 2022; Loo, 2016
				REML + classic	-1.10 (-2.91, 0.72)	0.236	0.0	
	Cranberry**	1	40	REML + HKSJ	0.00 (-1.96, 1.96)	1.000	NA	Richter, 2021
				REML + classic	0.00 (-1.96, 1.96)	1.000	NA	
**LDL Cholesterol (mg/dL)**								
Overall	Parallel RCT + Cross over RCT (r = 0.50)	3	180	REML + HKSJ	2.51 (-6.66, 11.67)	0.360	0.0	Woolf, 2023; Le Sayec, 2022; Richter, 2021
				REML + classic	2.51 (-2.22, 7.23)	0.299	0.0	
	Parallel RCT	2	140	REML + HKSJ	0.29 (-86.57, 87.16)	0.973	26.3	Woolf, 2023; Le Sayec, 2022
				REML + classic	0.29 (-13.11, 13.69)	0.966	26.3	
	Cross over RCT (r = 0.50)	1	40	REML + HKSJ	3.00 (-2.19, 8.19)	0.257	NA	Richter, 2021
				REML + classic	3.00 (-2.19, 8.19)	0.257	NA	

Group / Subgroup	Analysis specification	Studies (k)	Participants (N)	RE Method	Mean Difference (95% CI)	p-value	I^2	Included studies
Subgroup: Duration	> 8 weeks	2	140	REML + HKSJ	0.29 (-86.57, 87.16)	0.973	26.3	Woolf, 2023; Le Sayec, 2022
				REML + classic	0.29 (-13.11, 13.69)	0.966	26.3	
	≤ 8 weeks*	2	77	REML + HKSJ	3.00 (-2.19, 8.19)	0.257	NA	Richter, 2021
				REML + classic	3.00 (-2.19, 8.19)	0.257	NA	
**Triglycerides (mg/dL)**								
Overall	Parallel RCT + Cross over RCT (r = 0.50)	4	217	REML + HKSJ	-2.58 (-9.26, 4.09)	0.305	15.9	Loo, 2016; Richter, 2021; Woolf, 2023; Le Sayec, 2022
				REML + classic	-2.58 (-6.35, 1.18)	0.179	15.9	
	Parallel RCT	2	140	REML + HKSJ	-0.06 (-141.66, 141.54)	0.997	56.8	Woolf, 2023; Le Sayec, 2022
				REML + classic	-0.06 (-21.90, 21.78)	0.996	56.8	
	Cross over RCT (r = 0.50)	2	77	REML + HKSJ	-3.15 (-18.36, 12.05)	0.231	0.0	Loo, 2016; Richter, 2021
				REML + classic	-3.15 (-7.10, 0.79)	0.117	0.0	
Subgroup: Duration	> 8 weeks	2	140	REML + HKSJ	-0.06 (-141.66, 141.54)	0.997	56.8	Woolf, 2023; Le Sayec, 2022
				REML + classic	-0.06 (-21.90, 21.78)	0.996	56.8	
	≤ 8 weeks**	2	77	REML + HKSJ	-3.15 (-18.36, 12.05)	0.231	0.0	Loo, 2016; Richter, 2021
				REML + classic	-3.15 (-7.10, 0.79)	0.117	0.0	
Subgroup: Supplement presentation	Freeze-dried powder	1	43	REML + HKSJ	-14.00 (-39.57, 11.57)	0.283	NA	Woolf, 2023
				REML + classic	-14.00 (-39.57, 11.57)	0.283	NA	
	Capsule	1	97	REML + HKSJ	8.85 (-5.71, 23.41)	0.234	NA	Le Sayec, 2022
				REML + classic	8.85 (-5.71, 23.41)	0.234	NA	
	Juice**	2	77	REML + HKSJ	-3.15 (-18.36, 12.05)	0.231	0.0	Loo, 2016; Richter, 2021
				REML + classic	-3.15 (-7.10, 0.79)	0.117	0.0	
Subgroup: Berry type	Blueberry	1	43	REML + HKSJ	-14.00 (-39.57, 11.57)	0.283	NA	Woolf, 2023
				REML + classic	-14.00 (-39.57, 11.57)	0.283	NA	
	Chokeberry	2	134	REML + HKSJ	0.91 (-66.64, 68.45)	0.892	54.7	Le Sayec, 2022; Loo, 2016
				REML + classic	0.91 (-9.51, 11.33)	0.864	54.7	
	Cranberry*	1	40	REML + HKSJ	-6.00 (-16.18, 4.18)	0.248	NA	Richter, 2021
				REML + classic	-6.00 (-16.18, 4.18)	0.248	NA	

REML: Restricted Maximum Likelihood; HKSJ: Hartung-Knapp-Sidik-Jonkman; NA: Not applicable

For parallel RCT with two intervention arms compilation in one single arm was performed as recommended by Cochrane manual (Chapter 6; 6.5.2.10) using sd: $\sqrt{[[(n1-1)\cdot sd1^2 + (n2-1)\cdot sd2^2 + (n1\cdot n2/(n1+n2))\cdot(mean1^2 + mean2^2 - 2\cdot mean1\cdot mean2)] / (n1+n2-1)]}$

Mean difference was calculated for cross-over RCT using correlation coefficients 0.5, 0.25 and 0.75 as recommended by Cochrane manual (Chapter 6; 6.5.2.10) using $(sd\_treat^2 + sd\_cont^2 - 2\cdot r\cdot sd\_treat\cdot sd\_cont)$

Parallel RCT with 3 arms: Tjelle 2015 and Jeong, 2015

*For subgroup analysis all cross-over RCT were included considering a correlation of 0.5 for paired differences

**For subgroup analysis only Richter, 2021 cross-over RCT was included considering a correlation of 0.5 for paired differences. For the rest of cross-over RCT the reported mean difference was used

Supplementary Table S3. Inflammatory outcomes.

Group / Subgroup	Analysis specification	Studies (k)	Participants (N)	RE Method	Mean Difference (95% CI)	p-value	I^2	Included studies
**C Reactive Protein (mg/dL)**								
Overall	Parallel RCT + Cross over RCT (r = 0.50)	3	125	REML + HKSJ	0.00 (-0.01, 0.01)	0.864	0.0	Richter, 2021; Johnson 2015 & Johnson 2017; Jeong, 2015
				REML + classic	0.00 (-0.01, 0.01)	0.876	0.0	
	Parallel RCT	2	85	REML + HKSJ	0.00 (-0.01, 0.01)	0.983	0.0	Johnson 2015 & Johnson 2017; Jeong, 2015
				REML + classic	0.00 (-0.01, 0.01)	0.996	0.0	
	Cross over RCT (r = 0.50)	1	40	REML + HKSJ	0.03 (-0.02, 0.08)	0.257	NA	Richter, 2021
				REML + classic	0.03 (-0.02, 0.08)	0.257	NA	
**Tumor Necrosis Factor-alfa (pg/mL)**								
Overall	Parallel RCT + Cross over RCT (r = 0.50)	2	77	REML + HKSJ	0.30 (-13.06, 13.66)	0.824	89.4	Loo, 2016; Johnson 2015 & Johnson 2017
				REML + classic	0.30 (-1.76, 2.36)	0.777	89.4	

REML: Restricted Maximum Likelihood; HKSJ: Hartung-Knapp-Sidik-Jonkman; NA: Not applicable

For parallel RCT with two intervention arms compilation in one single arm was performed as recommended by Cochrane manual (Chapter 6; 6.5.2.10) using sd: $\sqrt{[[(n1-1)\cdot sd1^2 + (n2-1)\cdot sd2^2 + (n1\cdot n2/(n1+n2))\cdot(mean1^2 + mean2^2 - 2\cdot mean1\cdot mean2)] / (n1+n2-1)]}$

Mean difference was calculated for cross-over RCT using correlation coefficients 0.5, 0.25 and 0.75 as recommended by Cochrane manual (Chapter 6; 6.5.2.10) using $(sd\_treat^2 + sd\_cont^2 - 2\cdot r\cdot sd\_treat\cdot sd\_cont)$

Figure S1. Augmentation index standardized at 75 beats per minute (%) forest plot

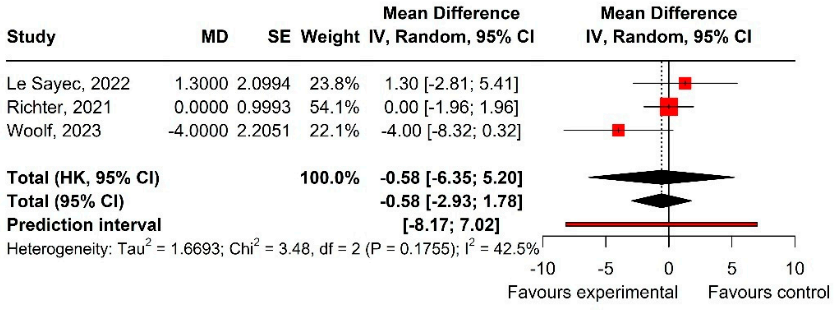

Note: For Richter, 2021, effect calculated assuming r=0.5 (cross-over)

Figure S2. Flow mediated dilation (%) forest plot

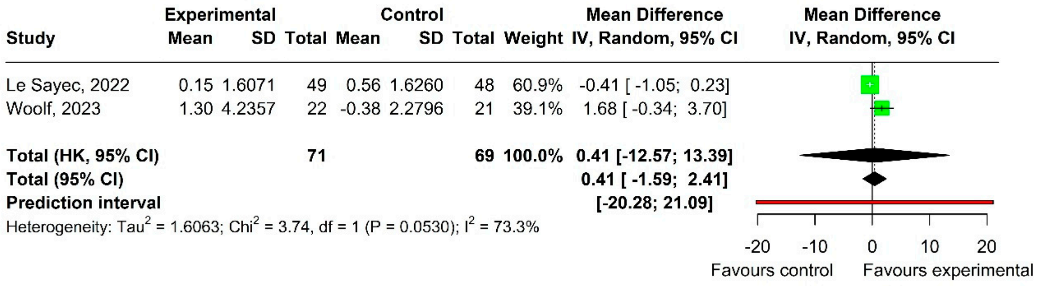

Figure S3. Pulse wave velocity: carotid–femoral pulse wave velocity (m/s) forest plot

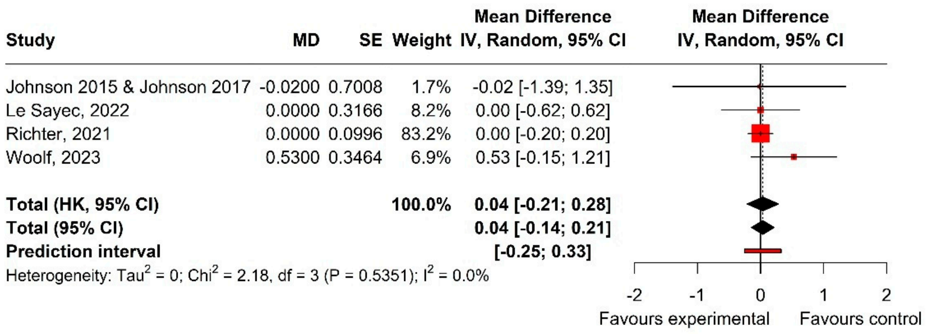

Note: For Richter, 2021, effect calculated assuming r=0.5 (cross-over)

Figure S4. Pulse wave velocity: brachial–aortic pulse wave velocity (m/s) forest plot

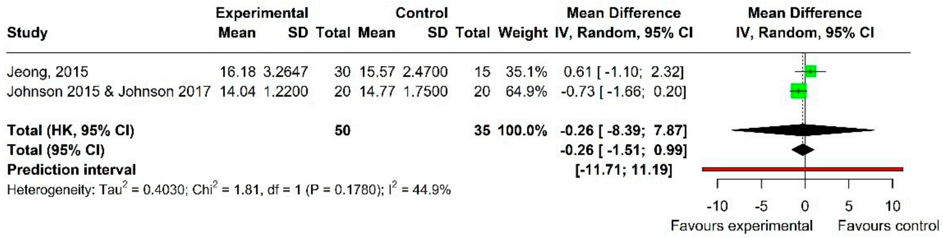

Note: For Jeong, 2015 experimental arms were combined

Figure S5. Total cholesterol (mg/dL) forest plot

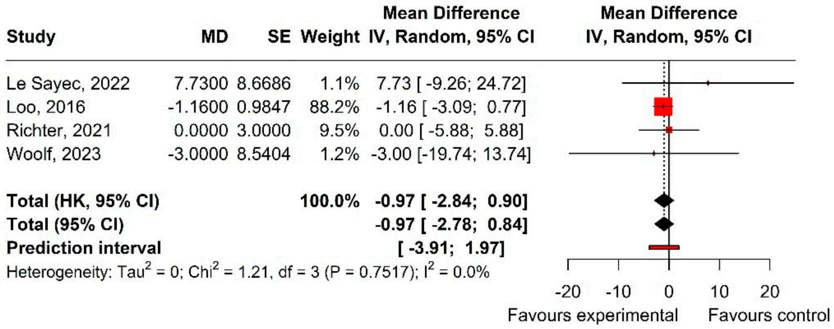

Note: For Richter, 2021 and Loo, 2016 effects are calculated assuming r=0.5 (cross-over)

Figure S6. HDL cholesterol (mg/dL) forest plot

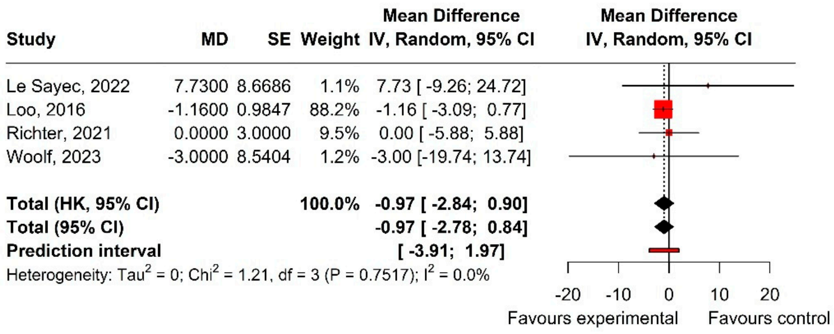

Note: For Richter, 2021 and Loo, 2016 effects are calculated assuming r=0.5 (cross-over)

Figure S7. LDL cholesterol (mg/dL) forest plot

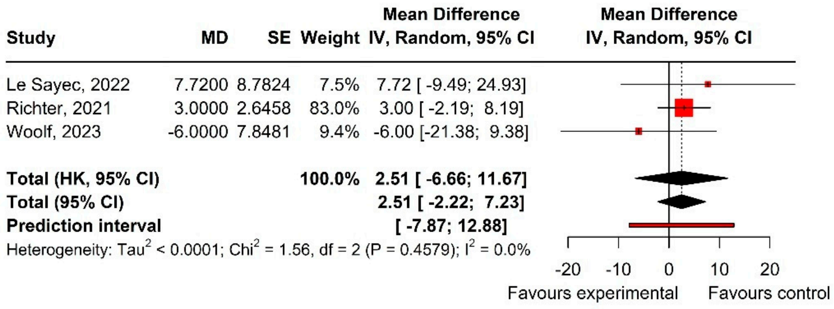

Note: For Richter, 2021 effects are calculated assuming r=0.5 (cross-over)

Figure S8. Triglycerides (mg/dL) forest plot

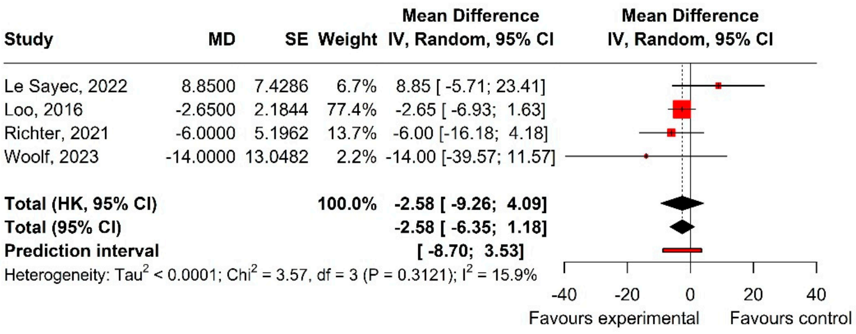

Note: For Richter, 2021 effects are calculated assuming r=0.5 (cross-over)

Figure S9. C-reactive protein (mg/dL) forest plot

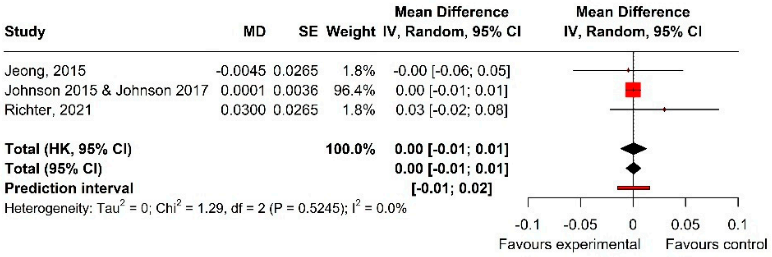

Note: For Richter, 2021 effects are calculated assuming r=0.5 (cross-over)

Figure S10. Tumor necrosis factor alpha (TNF-α) (pg/mL) forest plot

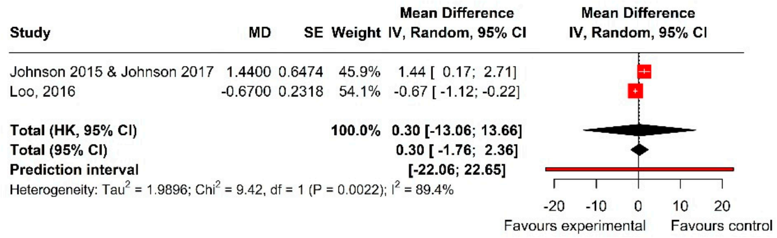

Figure S11. Systolic blood pressure (mmHg) sensitivity analysis (r = 0.25) forest plot

Study	MD	SE	Weight	Mean Difference IV, Random, 95% CI
Kianbakhta, 2019	-10.3000	2.0049	13.2%	-10.30 [-14.23; -6.37]
Johnson 2015 & Johnson 2017	-8.0000	5.0695	5.2%	-8.00 [-17.94; 1.94]
Loo, 2016	-2.3000	1.6519	14.5%	-2.30 [-5.54; 0.94]
Jeong, 2015	-2.2000	4.3592	6.4%	-2.20 [-10.74; 6.34]
Richter, 2021	-2.0000	1.2247	16.0%	-2.00 [-4.40; 0.40]
Le Sayec, 2022	-1.0000	1.6652	14.4%	-1.00 [-4.26; 2.26]
Keane, 2016	0.0000	3.6742	7.9%	0.00 [-7.20; 7.20]
Tjelle, 2015	0.1069	2.3188	12.0%	0.11 [-4.44; 4.65]
Woolf, 2023	3.0000	2.8284	10.3%	3.00 [-2.54; 8.54]
**Random effects model (HK)**			**100.0%**	**-2.39 [-5.49; 0.70]**
**Random effects (classic, 95% CI)**				**-2.39 [-5.08; 0.30]**
**Prediction interval**				**[-10.43; 5.65]**

Heterogeneity: $Tau^2 = 10.2700$; $Chi^2 = 22.81$, df = 8 (P = 0.0036); $I^2 = 64.9\%$

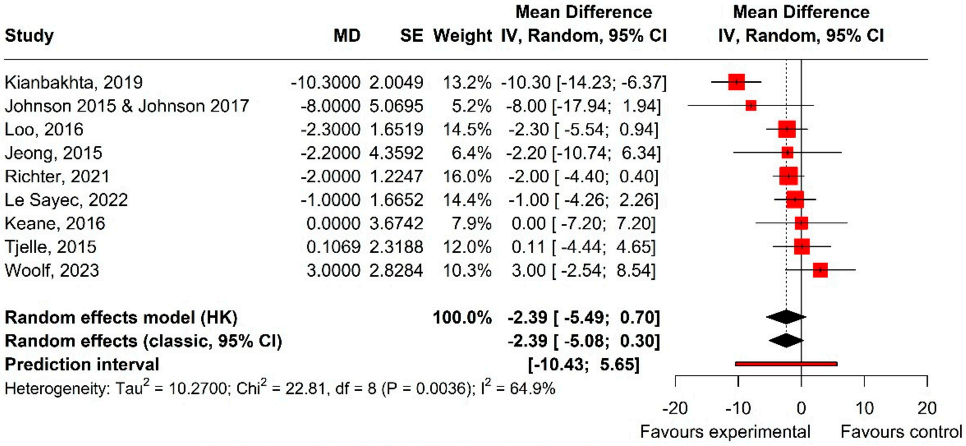

Note: For Keane, 2016 and Richter, 2021, effect calculated assuming r=0.25 (cross-over)

Figure S12. Systolic blood pressure (mmHg) sensitivity analysis (r = 0.75) forest plot

Study	MD	SE	Weight	Mean Difference IV, Random, 95% CI
Kianbakhta, 2019	-10.3000	2.0049	12.4%	-10.30 [-14.23; -6.37]
Johnson 2015 & Johnson 2017	-8.0000	5.0695	4.8%	-8.00 [-17.94; 1.94]
Loo, 2016	-2.3000	1.6519	13.7%	-2.30 [-5.54; 0.94]
Jeong, 2015	-2.2000	4.3592	5.9%	-2.20 [-10.74; 6.34]
Richter, 2021	-2.0000	0.7071	16.6%	-2.00 [-3.39; -0.61]
Le Sayec, 2022	-1.0000	1.6652	13.6%	-1.00 [-4.26; 2.26]
Keane, 2016	0.0000	2.1213	12.0%	0.00 [-4.16; 4.16]
Tjelle, 2015	0.1069	2.3188	11.3%	0.11 [-4.44; 4.65]
Woolf, 2023	3.0000	2.8284	9.6%	3.00 [-2.54; 8.54]
**Random effects model (HK)**			**100.0%**	**-2.28 [-5.30; 0.75]**
**Random effects (classic, 95% CI)**				**-2.28 [-4.84; 0.29]**
**Prediction interval**				**[-10.10; 5.55]**

Heterogeneity: $Tau^2 = 9.7946$; $Chi^2 = 23.68$, df = 8 (P = 0.0026); $I^2 = 66.2\%$

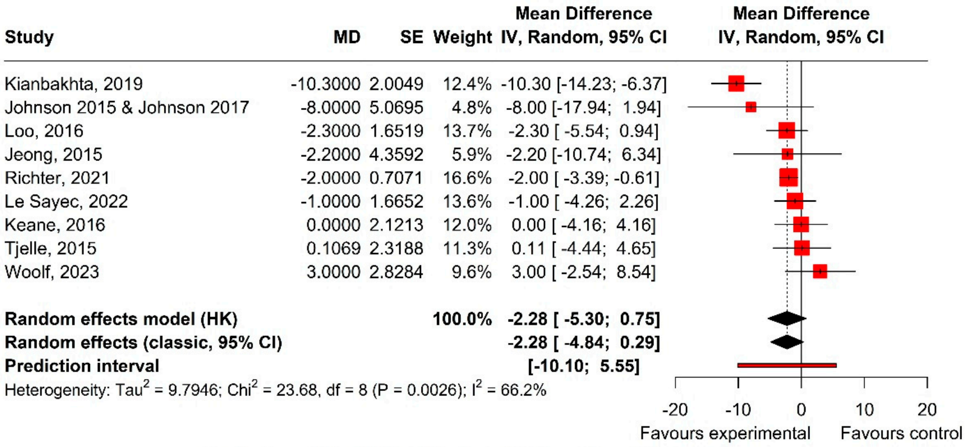

Note: For Keane, 2016 and Richter, 2021, effect calculated assuming r=0.75 (cross-over)

Figure S13. Diastolic blood pressure (mmHg) sensitivity analysis (r = 0.25) forest plot

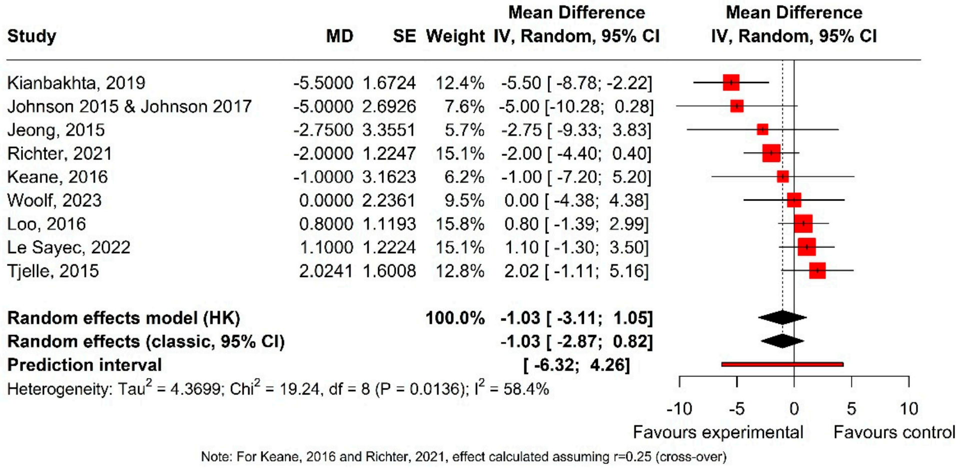

Figure S14. Diastolic blood pressure (mmHg) sensitivity analysis (r = 0.75) forest plot

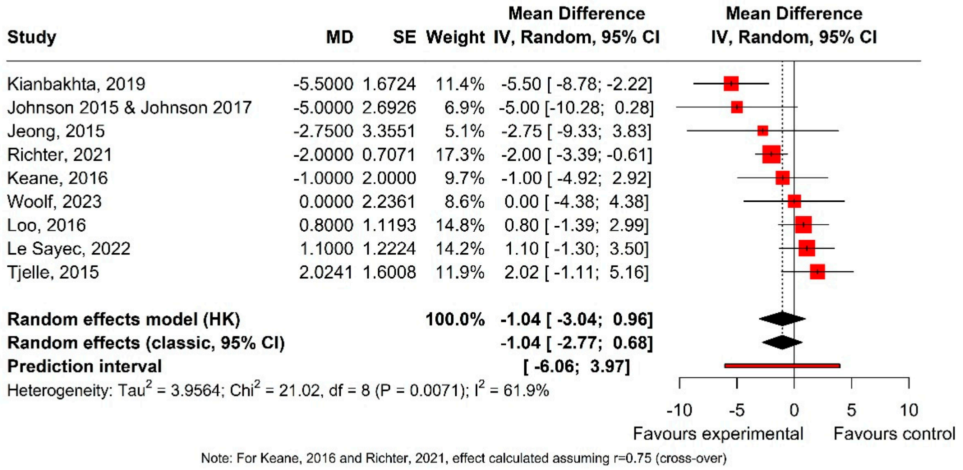

Figure S15. 24-hour systolic blood pressure (mmHg) sensitivity analysis (r = 0.25) forest plot

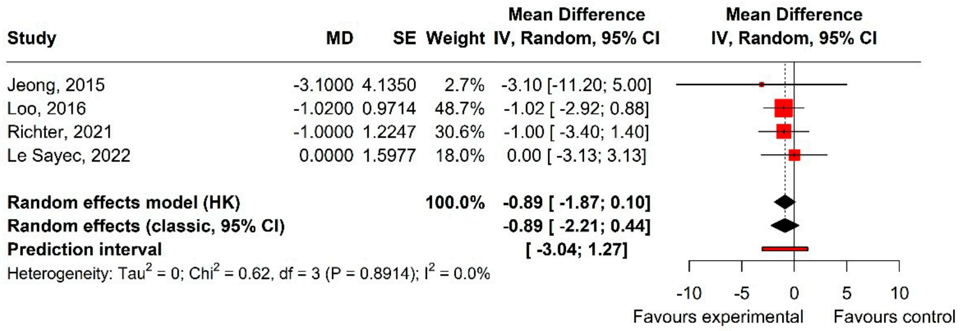

Note: For Jeong, 2015 experimental arms were combined. For Richter, 2021 effect calculated assumming r=0.25 (cross-over)

Figure S16. 24-hour systolic blood pressure (mmHg) sensitivity analysis (r = 0.75) forest plot

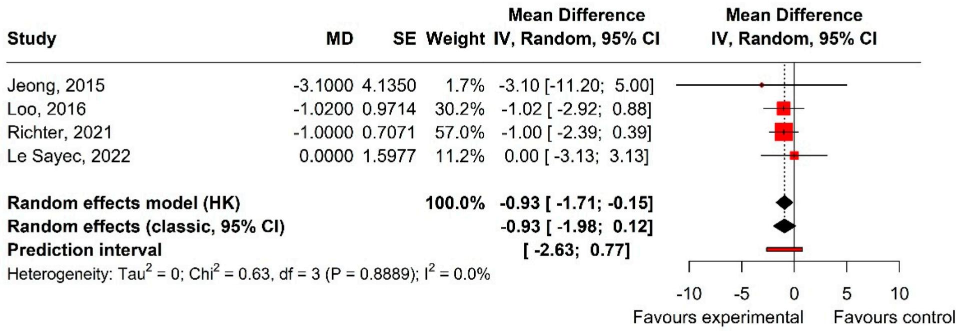

Note: For Jeong, 2015 experimental arms were combined. For Richter, 2021 effect calculated assumming r=0.75 (cross-over)

Figure S17. 24-hour diastolic blood pressure (mmHg) sensitivity analysis (r = 0.25) forest plot

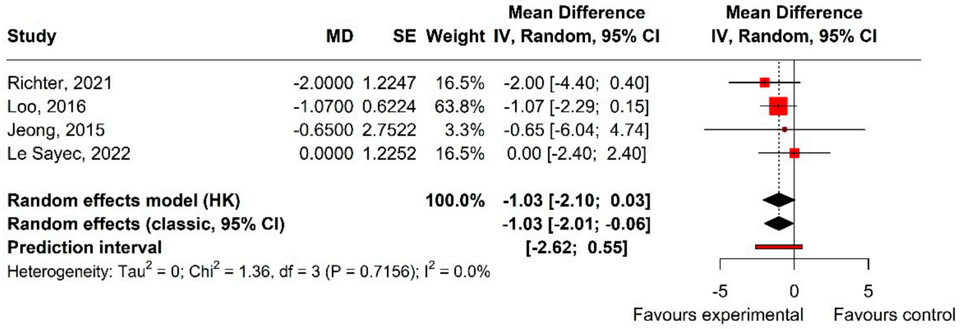

Note: For Jeong, 2015 experimental arms were combined. For Richter, 2021 effect calculated assumming r=0.25 (cross-over)

Figure S18. 24-hour diastolic blood pressure (mmHg) sensitivity analysis (r = 0.75) forest plot

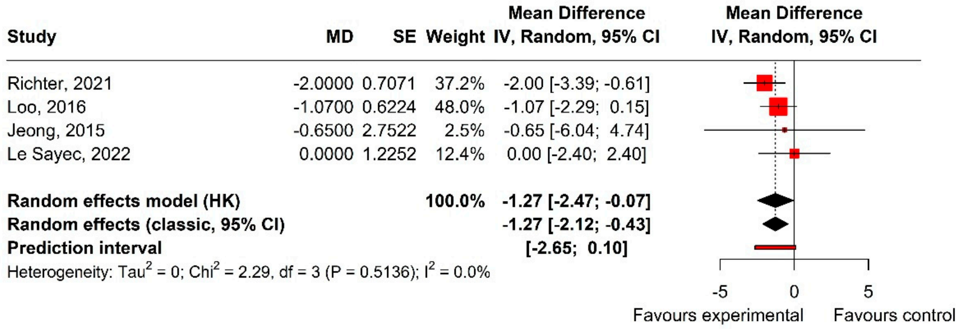

Note: For Jeong, 2015 experimental arms were combined. For Richter, 2021 effect calculated assumming r=0.75 (cross-over)

Figure S19. Augmentation Index at 75 bpm (%) sensitivity analysis (r = 0.25) forest plot

Study	MD	SE	Weight	Mean Difference IV, Random, 95% CI
Le Sayec, 2022	1.3000	2.0994	26.3%	1.30 [-2.81; 5.41]
Richter, 2021	0.0000	1.2239	49.1%	0.00 [-2.40; 2.40]
Woolf, 2023	-4.0000	2.2051	24.6%	-4.00 [-8.32; 0.32]
**Total (HK, 95% CI)**			**100.0%**	**-0.64 [-6.70; 5.42]**
**Total (95% CI)**				**-0.64 [-3.16; 1.88]**
**Prediction interval**				**[-8.72; 7.44]**

Heterogeneity: $Tau^2 = 1.8733$; $Chi^2 = 3.42$, df = 2 (P = 0.1808); $I^2 = 41.5\%$

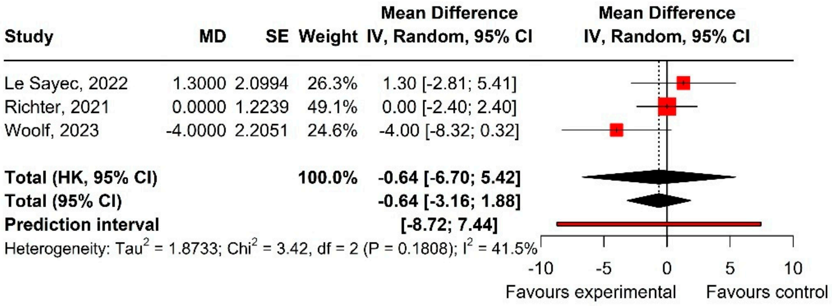

Note: For Richter, 2021, effect calculated assuming r=0.25 (cross-over)

Figure S20. Augmentation Index at 75 bpm (%) sensitivity analysis (r = 0.75) forest plot

Study	MD	SE	Weight	Mean Difference IV, Random, 95% CI
Le Sayec, 2022	1.3000	2.0994	20.3%	1.30 [-2.81; 5.41]
Richter, 2021	0.0000	0.7066	60.9%	0.00 [-1.38; 1.38]
Woolf, 2023	-4.0000	2.2051	18.8%	-4.00 [-8.32; 0.32]
**Total (HK, 95% CI)**			**100.0%**	**-0.49 [-5.86; 4.88]**
**Total (95% CI)**				**-0.49 [-2.63; 1.65]**
**Prediction interval**				**[-7.49; 6.51]**

Heterogeneity: $Tau^2 = 1.4557$; $Chi^2 = 3.56$, df = 2 (P = 0.1687); $I^2 = 43.8\%$

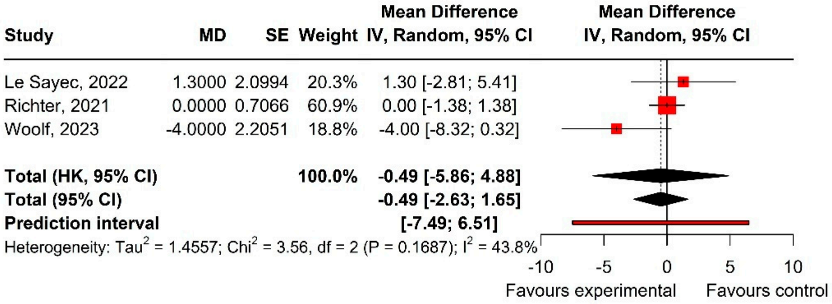

Note: For Richter, 2021, effect calculated assuming r=0.75 (cross-over)

Figure S21. Carotid-femoral pulse wave velocity (m/s) sensitivity analysis (r = 0.25) forest plot

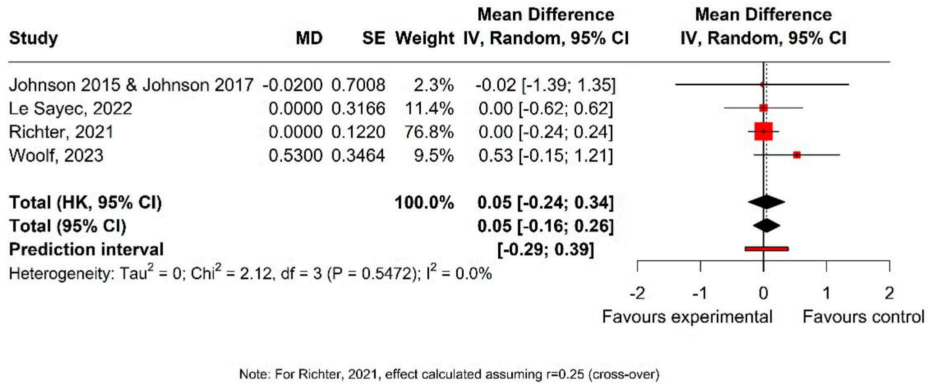

Figure S22. Carotid-femoral pulse wave velocity (m/s) sensitivity analysis (r = 0.75) forest plot

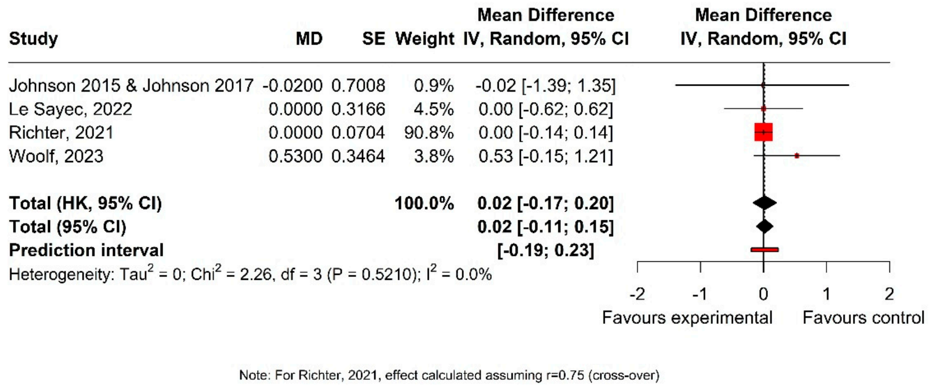

Figure S23. Total cholesterol (mg/dL) sensitivity analysis (r = 0.25) forest plot

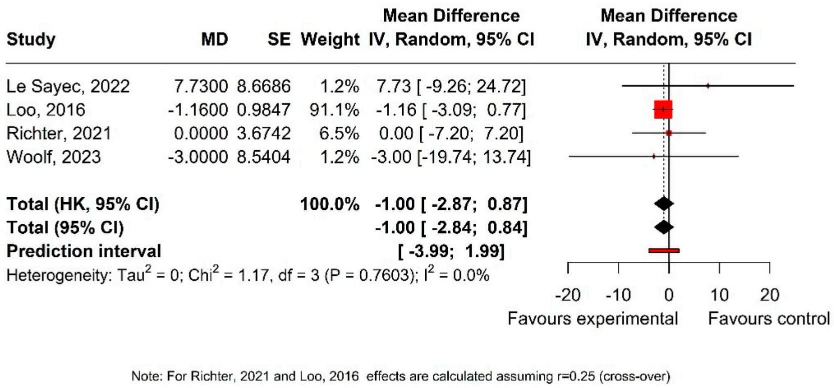

Figure S24. Total cholesterol (mg/dL) sensitivity analysis (r = 0.75) forest plot

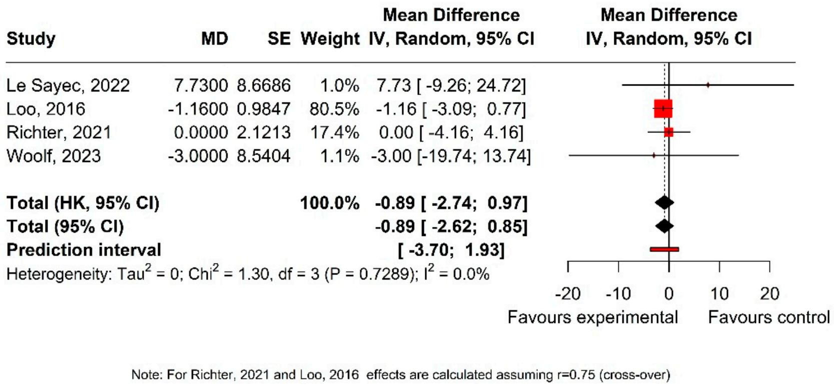

Figure S25. HDL cholesterol (mg/dL) sensitivity analysis (r = 0.25) forest plot

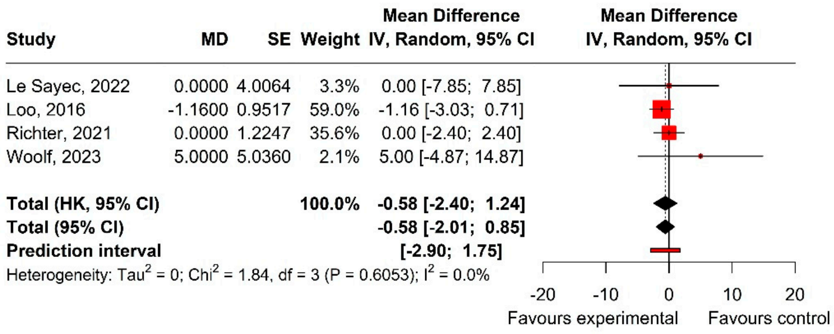

Note: For Richter, 2021 effects are calculated assuming r=0.25 (cross-over)

Figure S26. HDL cholesterol (mg/dL) sensitivity analysis (r = 0.75) forest plot

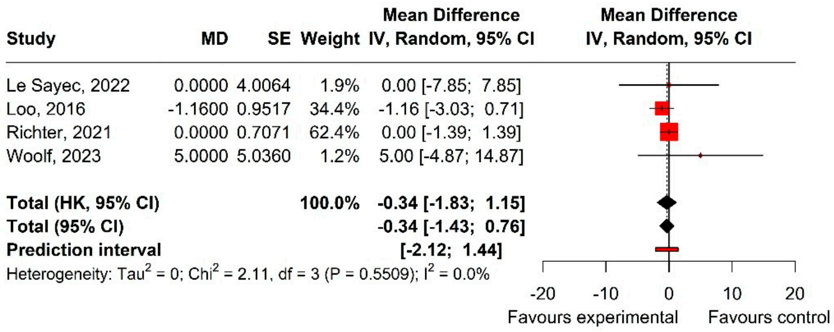

Note: For Richter, 2021 effects are calculated assuming r=0.75 (cross-over)

Figure S27. LDL cholesterol (mg/dL) sensitivity analysis (r = 0.25) forest plot

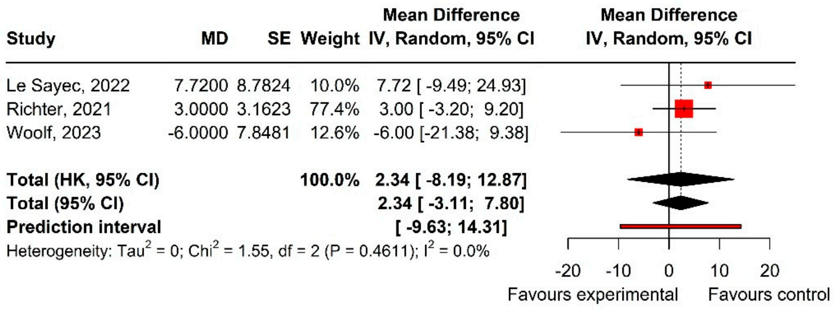

Note: For Richter, 2021 effects are calculated assuming r=0.25 (cross-over)

Figure S28. LDL cholesterol (mg/dL) sensitivity analysis (r = 0.75) forest plot

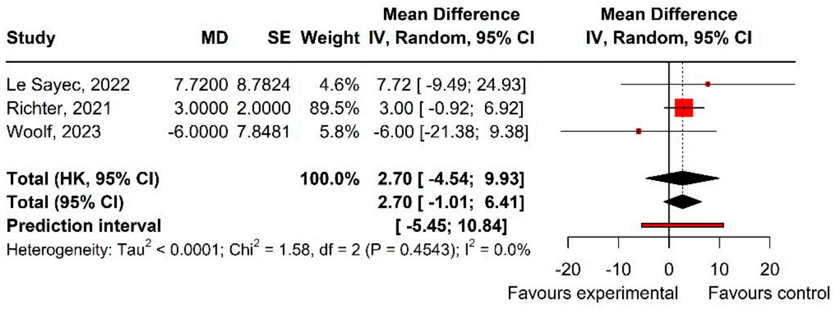

Note: For Richter, 2021 effects are calculated assuming r=0.75 (cross-over)

Figure S29. Triglycerides (mg/dL) sensitivity analysis (r = 0.25) forest plot

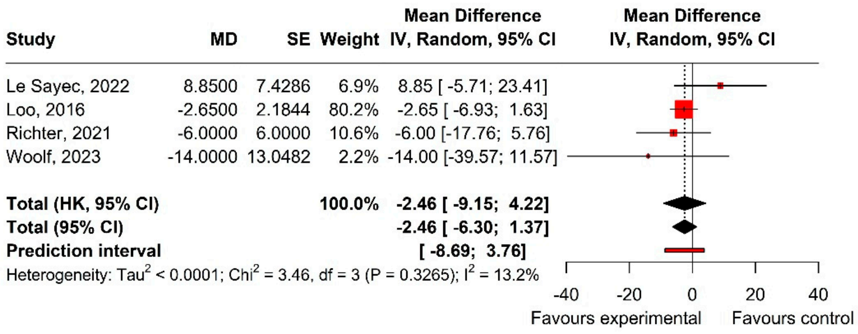

Note: For Richter, 2021 effects are calculated assuming r=0.25 (cross-over)

Figure S30. Triglycerides (mg/dL) sensitivity analysis (r = 0.75) forest plot

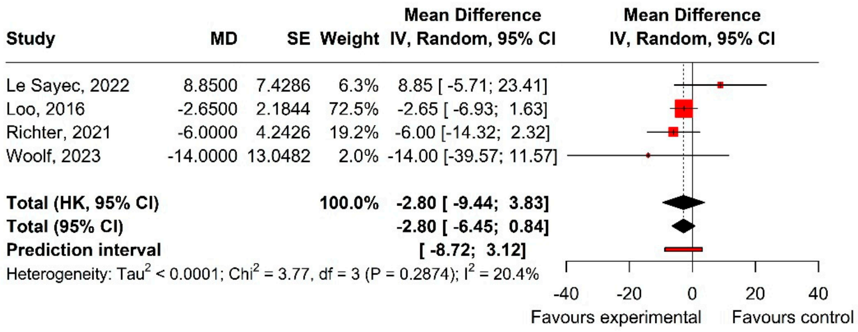

Note: For Richter, 2021 effects are calculated assuming r=0.75 (cross-over)

Figure S31. C-reactive protein (mg/dL) sensitivity analysis (r = 0.25) forest plot

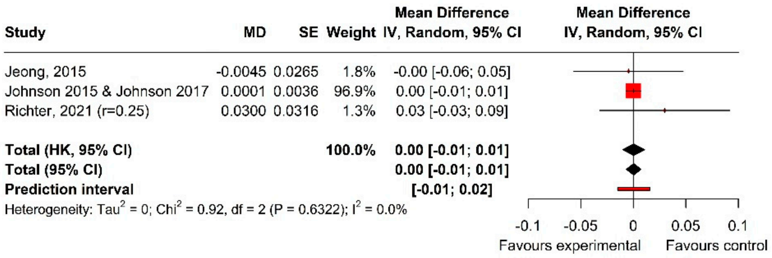

Note: For Richter, 2021 effects are calculated assuming r=0.25 (cross-over)

Figure S32. C-reactive protein (mg/dL) sensitivity analysis (r = 0.75) forest plot

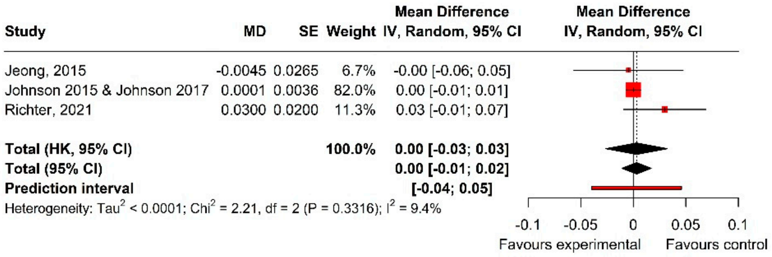

Note: For Richter, 2021 effects are calculated assuming r=0.75 (cross-over)

Supplementary Table S4. GRADE certainty of evidence of additional outcomes

Berry compared to Placebo for blood pressure, vascular and metabolic outcomes in pre-hypertension and hypertension

Patient or population: Pre-hypertension and hypertension

Setting: Community

Intervention: Berry

Comparison: Placebo

Outcomes	Anticipated absolute effects[*] (95% CI)		Relative effect (95% CI)	№ of participants (studies)	Certainty of the evidence (GRADE)	Comments
	Risk with Placebo	Risk with Berry				
Augmentation Index at 75 bpm (Aix75) follow-up: range 8 weeks to 12 weeks	The mean augmentation Index at 75 bpm (AIx75) was **0** %	MD **0.58 % lower** (2.93 lower to 1.78 higher)	-	180 (3 RCTs)[1,2,3]	⊕⊕◯◯ Low[a,b,c,d]	Berry may result in little to no difference in augmentation Index at 75 bpm (AIx75).
Flow-mediated dilation (FMD) (FMD) follow-up: 12 weeks	The mean flow-mediated dilation (FMD) was **0** %	MD **0.41 % higher** (1.59 lower to 2.41 higher)	-	140 (2 RCTs)[1,2]	⊕◯◯◯ Very low[d,e,f,g]	The evidence is very uncertain about the effect of Berry on flow-mediated dilation (FMD) .
Carotid-femoral pulse wave velocity (m/s) (PWV(m/s) carotid) follow-up: range 8 weeks to 12 weeks	The mean carotid-femoral pulse wave velocity (m/s) was **0** m/s	MD **0.04 m/s higher** (0.14 lower to 0.21 higher)	-	220 (4 RCTs)[1,2,3,4]	⊕⊕◯◯ Low[d,h,i,j]	Berry may result in little to no difference in carotid-femoral pulse wave velocity (m/s).
Brachial-aortic pulse wave velocity (m/s) (PWV (m/s) brachial) follow-up: 8 weeks	The mean brachial-aortic pulse wave velocity (m/s) was **0** m/s	MD **0.26 m/s lower** (1.51 lower to 0.99 higher)	-	85 (2 RCTs)[4,5]	⊕◯◯◯ Very low[k,l,m,n]	The evidence is very uncertain about the effect of Berry on brachial-aortic pulse wave velocity (m/s).
Total Cholesterol (mg/dL) (Cholesterol) follow-up: range 8 weeks to 12 weeks	The mean Total Cholesterol (mg/dL) was **0** mg/dL	MD **0.11 mg/dL higher** (4.58 lower to 4.8 higher)	-	217 (4 RCTs)[1,2,3,6]	⊕◯◯◯ Very low[o,p,q,r]	The evidence is very uncertain about the effect of Berry on Total Cholesterol (mg/dL).

**Berry compared to Placebo for blood pressure, vascular and metabolic outcomes in pre-hypertension and hypertension**						
**Patient or population:** Pre-hypertension and hypertension<br>**Setting:** Community<br>**Intervention:** Berry<br>**Comparison:** Placebo						
Outcomes	**Anticipated absolute effects*** (95% CI)		Relative effect (95% CI)	№ of participants (studies)	Certainty of the evidence (GRADE)	Comments
	**Risk with Placebo**	**Risk with Berry**				
LDL Cholesterol (mg/dL) (LDL-c) follow-up: range 8 weeks to 12 weeks	The mean LDL Cholesterol (mg/dL) was **0** mg/dL	MD **2.51 mg/dL higher** (2.22 lower to 7.23 higher)	-	180 (3 RCTs)[1,2,3]	⊕⊕◯◯ Low[d,h,s,t]	Berry may result in little to no difference in LDL Cholesterol (mg/dL).
HDL Cholesterol (mg/dL) (HDL-c) follow-up: range 8 weeks to 12 weeks	The mean HDL Cholesterol (mg/dL) was **0** mg/dL	MD **0.49 mg/dL lower** (1.81 lower to 0.83 higher)	-	217 (4 RCTs)[1,2,3,6]	⊕◯◯◯ Very low[o,r,u,v]	The evidence is very uncertain about the effect of Berry on HDL Cholesterol (mg/dL).
Triglycerides (mg/dL) (TG) follow-up: range 8 weeks to 12 weeks	The mean Triglycerides (mg/dL) was **0** md/dL	MD **2.58 md/dL lower** (6.35 lower to 1.18 higher)	-	217 (4 RCTs)[1,2,3,6]	⊕◯◯◯ Very low[o,r,w,x]	The evidence is very uncertain about the effect of Berry on Triglycerides (mg/dL).
C Reactive Protein (mg/dL) (CRP) follow-up: range 8 weeks to 12 weeks	The mean C Reactive Protein (mg/dL) was **0** mg/dL	MD **0 mg/dL** (0.01 lower to 0.01 higher)	-	125 (3 RCTs)[3,5,7]	⊕⊕◯◯ Low[aa,ab,y,z]	Berry may result in little to no difference in C Reactive Protein (mg/dL).
Tumor Necrosis Factor-alfa (pg/mL) follow-up: 8 weeks	The mean TNF-alfa (pg/mL) was **0** pg/mL	MD **0.3 pg/mL higher** (1.76 lower to 2.36 higher)	-	77 (2 RCTs)[6,7]	⊕◯◯◯ Very low[ac,ad,ae,n,o]	The evidence is very uncertain about the effect of Berry on TNF-alfa (pg/mL).
***The risk in the intervention group** (and its 95% confidence interval) is based on the assumed risk in the comparison group and the **relative effect** of the intervention (and its 95% CI).<br>**CI:** confidence interval; **MD:** mean difference						

Berry compared to Placebo for blood pressure, vascular and metabolic outcomes in pre-hypertension and hypertension

Patient or population: Pre-hypertension and hypertension

Setting: Community

Intervention: Berry

Comparison: Placebo

Outcomes	**Anticipated absolute effects*** (95% CI)		Relative effect (95% CI)	№ of participants (studies)	Certainty of the evidence (GRADE)	Comments
	**Risk with Placebo**	**Risk with Berry**				

GRADE Working Group grades of evidence
High certainty: we are very confident that the true effect lies close to that of the estimate of the effect.
Moderate certainty: we are moderately confident in the effect estimate: the true effect is likely to be close to the estimate of the effect, but there is a possibility that it is substantially different.
Low certainty: our confidence in the effect estimate is limited: the true effect may be substantially different from the estimate of the effect.
Very low certainty: we have very little confidence in the effect estimate: the true effect is likely to be substantially different from the estimate of effect.

Explanations

a. Included studies judged as low risk of bias correspond to more than 75% of weight in meta-analysis

b. Moderate heterogeneity (I^2=42.5%; P value [p = 0.175]). Effect estimates with overlapping confidence intervals. No evidence of opposing effects.

c. Serious imprecision. Confidence interval does not cross imprecision threshold (defined as 0.2*SD. This corresponds to 3%) but the optimal information size (OIS, defined as n = 400 following GRADE guidelines) was not met

d. All the included studies in the meta-analysis were fully or partially funded by private organizations or industries related to the berry market

e. Small number of studies included. However, the low risk of bias study included correspond to 60.9% of weight in meta-analysis

f. Substantial heterogeneity (I^2 = 73.3 %; P value [p = 0.053]) with opposite directions of effect across the two studies.

g. Very serious imprecision. Confidence interval crossed two imprecision thresholds (defined as 0.2*SD. This corresponds to 0.46%)

h. Included studies judged as low risk of bias correspond to more than 90% of weight in meta-analysis

i. Not important heterogeneity (I^2=0%; P value [p = 0.535]). Effect estimates with overlapping confidence intervals. No evidence of opposing effects.

j. Serious imprecision. Confidence interval does not cross imprecision threshold (defined as 0.2*SD. This corresponds to 0.6 m/s) but the optimal information size (OIS, defined as n = 400 following GRADE guidelines) was not met

k. Small number of studies included. The study judged as low risk of bias correspond to 35.1% of weight in meta-analysis. Point estimates direction differed between the low and the unclear risk of bias.

l. Moderate heterogeneity (I^2=44.9%; P value [p = 0.178]). The point estimates differed in direction between studies, suggesting true inconsistency. Small number of studies.

m. Very serious imprecision. Confidence interval crossed two imprecision thresholds (defined as 0.2*SD. This corresponds to 0.49 m/s)

n. Due to Johnson 2015 & Johnson 2017 was funded by private organizations related to the berry market

o. Due to unclear blinding in Loo, 2016 and lack of wash-out period lowering confidence in the estimated effect.

p. Not important heterogeneity (I^2=0%; P value [p = 0.809]). Effect estimates with overlapping confidence intervals. No evidence of opposing effects.

q. Serious imprecision. Confidence interval does not cross imprecision threshold (defined as 0.2*SD. This corresponds to 13 mg/dL) but the optimal information size (OIS, defined as n = 400 following GRADE guidelines) was not met

r. Three out of the four studies included in the meta-analysis were fully or partially funded by private organizations or industries related to the berry market

s. Not important heterogeneity (I^2=0%; P value [p = 0.458]). Effect estimates with overlapping confidence intervals. No evidence of opposing effects.

t. Serious imprecision. Confidence interval does not cross imprecision threshold (defined as 0.2*SD. This corresponds to 14 mg/dL) but the optimal information size (OIS, defined as n = 400 following GRADE guidelines) was not met

u. Not important heterogeneity (I^2=0%; P value [p = 0.768]). Effect estimates with overlapping confidence intervals. No evidence of opposing effects.

v. Serious imprecision. Confidence interval does not cross imprecision threshold (defined as 0.2*SD. This corresponds to 6 mg/dL) but the optimal information size (OIS, defined as n = 400 following GRADE guidelines) was not met

w. Not important heterogeneity (I^2=15.9%; P value [p = 0.312]). The point estimates differed in direction between studies, suggesting true inconsistency. Small number of studies.

x. Serious imprecision. Confidence interval does not cross imprecision threshold (defined as 0.2*SD. This corresponds to 13 mg/dL) but the optimal information size (OIS, defined as n = 400 following GRADE guidelines) was not met

y. Small number of studies included. The low risk of bias study included correspond to <50% of weight in meta-analysis. However, point estimates did not substantially differ between low and unclear risk of bias

z. Not important heterogeneity (I^2=0%; P value [p = 0.525]). Effect estimates with overleaping confidence intervals. No evidence of opposing effects.

aa. Serious imprecision. Confidence interval does not cross imprecision threshold (defined as 0.2*SD. This corresponds to 0.029 mg/dL) but the optimal information size (OIS, defined as n = 400 following GRADE guidelines) was not met

ab. Two out of the four included studies in the meta-analysis were fully or partially funded by private organizations or industries related to the berry market

ac. Small number of studies. Only unclear risk of bias studies available for meta-analysis.

ad. Considerable heterogeneity (I^2 = 89.4 %; P value [p = 0.002]) with opposite directions of effect across the two studies. Small number of studies.

ae. Very serious imprecision. Confidence interval crossed two imprecision thresholds (defined as 0.2*SD. This corresponds to 0.62 pg/mL)

References

1.Woolf, E. K.,J. D. Terwoord,N. S. Litwin,A. R. Vazquez,S. Y. Lee,N. Ghanem,K. A. Michell,B. T. Smith,L. E. Grabos,N. B. Ketelhut,N. P. Bachman,M. E. Smith,M. Le Sayec,S. Rao,C. L. Gentile,T. L. Weir,A. Rodriguez-Mateos,D. R. Seals,F. A. Dinenno,and S. A. Johnson.. Daily blueberry consumption for 12 weeks improves endothelial function in postmenopausal women with above-normal blood pressure through reductions in oxidative stress: a randomized controlled trial.Food Funct; 2023.

2.Le Sayec, M.,Y. Xu,M. Laiola,F. A. Gallego,D. Katsikioti,C. Durbidge,U. Kivisild,S. Armes,M. Lecomte,P. Fança-Berthon,E. Fromentin,F. Plaza Oñate,J. K. Cruickshank,and A. Rodriguez-Mateos. The effects of Aronia berry (poly)phenol supplementation on arterial function and the gut microbiome in middle aged men and women: Results from a randomized controlled trial.Clin Nutr; 2022.

3.Richter, C. K.,A. C. Skulas-Ray,T. L. Gaugler,S. Meily,K. S. Petersen,and P. M. Kris-Etherton. Effects of Cranberry Juice Supplementation on Cardiovascular Disease Risk Factors in Adults with Elevated Blood Pressure: A Randomized Controlled Trial.Nutrients; 2021.

4.Johnson, Sarah A.,Arturo Figueroa,Negin Navaei,Alexei Wong,Roy Kalfon,Lauren T. Ormsbee,Rafaela G. Feresin,Marcus L. Elam,Shirin Hooshmand,Mark E. Payton,and Bahram H. Arjmandi. Daily Blueberry Consumption Improves Blood Pressure and Arterial Stiffness in Postmenopausal Women with Pre- and Stage 1-Hypertension: A Randomized, Double-Blind, Placebo-Controlled Clinical Trial.Journal of the Academy of Nutrition and Dietetics; 2015.

5.Jeong, Han,Saem,Soon,Jun,Hong,Jae,Young,Cho,Tae-Bum,Lee,Ji-Wung,Kwon,Hyung,Joon,Joo,Jae,Hyoung,Park,Cheol,Woong,Yu,and,Do-Sun,Lim.. Effects of Rubus occidentalis extract on blood pressure in patients with prehypertension: Randomized, double-blinded, placebo-controlled clinical trial.Nutrition; 2016.

6.Loo, B. M.,I. Erlund,R. Koli,P. Puukka,J. Hellström,K. Wähälä,P. Mattila,and A. Jula. Consumption of chokeberry (Aronia mitschurinii) products modestly lowered blood pressure and reduced low-grade inflammation in patients with mildly elevated blood pressure.Nutr Res; 2016.

7.Johnson, S. A.,R. G. Feresin,N. Navaei,A. Figueroa,M. L. Elam,N. S. Akhavan,S. Hooshmand,S. Pourafshar,M. E. Payton,and B. H. Arjmandi. Effects of daily blueberry consumption on circulating biomarkers of oxidative stress, inflammation, and antioxidant defense in postmenopausal women with pre- and stage 1-hypertension: a randomized controlled trial.Food Funct; 2017.

Supplementary Table S5. GRADE evidence profile of main outcomes (resting systolic and diastolic blood pressure, and 24-hour systolic and diastolic blood pressure)

Certainty assessment							№ of patients		Effect		Certainty	Importance
**№ of studies**	**Study design**	**Risk of bias**	**Inconsistency**	**Indirectness**	**Imprecision**	**Other considerations**	**Berry**	**Placebo**	**Relative (95% CI)**	**Absolute (95% CI)**		
**Systolic blood pressure (mmHg)**												
9[1,2,3,4,5,6,7,8,9]	randomised trials	serious[a]	serious[b]	not serious	serious[c]	publication bias strongly suspected[d]	304	243	-	MD **2.35 mmHg lower** (4.98 lower to 0.29 higher)	⨁◯◯◯ Very low[a,b,c,d]	CRITICAL
**Diastolic blood pressure (mmHg)**												
9[1,2,3,4,5,6,7,8,9]	randomised trials	serious[a]	serious[e]	not serious	not serious[f]	publication bias strongly suspected[d]	304	243	-	MD **1.04 mmHg lower** (2.83 lower to 0.76 higher)	⨁◯◯◯ Very low[a,d,e,f]	CRITICAL
**24 hour- Systolic blood pressure (mmHg) (follow-up: range 8 weeks to 12 weeks)**												
4[1,2,3,9]	randomised trials	not serious[g]	not serious[h]	not serious	serious[i]	publication bias strongly suspected[j]	118	101	-	MD **0.90 mmHg lower** (2.14 lower to 0.34 higher)	⨁⨁◯◯ Low[g,h,i,j]	IMPORTANT
**24 hour- Diastolic blood pressure (mmHg) (follow-up: range 8 weeks to 12 weeks)**												
4[1,2,3,9]	randomised trials	not serious[g]	not serious[k]	not serious	serious[l]	publication bias strongly suspected[j]	118	101	-	MD **1.11 mmHg lower** (2.04 lower to 0.17 lower)	⨁⨁◯◯ Low[g,j,k,l]	IMPORTANT

CI: confidence interval; **MD:** mean difference

Explanations

a. Most of the meta-analysis weight correspond to trials which overall risk of bias were judged as "low" or "unclear"

b. Due to substantial heterogeneity (I^2=65%, P value [p=0.003]). The magnitude of the effect estimates varied considerably

c. Due to serious imprecision. Confidence interval crossed one imprecision threshold (defined as 0.2*SD. This corresponds to 4 mmHg)

d. Five out of the nine studies included in the meta-analysis were fully or partially funded by private organizations or industries related to the berry market

e. Due to moderate heterogeneity (I^2=56.6%, P value [p= 0.018]). Although the direction of effects was consistent across studies, the magnitude varied considerably, likely due to clinical and methodological diversity

f. Not serious imprecision. Confidence interval does not cross imprecision threshold (defined as 0.2*SD. This corresponds to 3 mmHg)

g. Most of the meta-analysis weight correspond to trials which overall risk of bias were judged as "low" or "unclear". However, point estimates did not substantially differ between "low" and "unclear" risk of bias studies

h. Not important heterogeneity (I^2=0%, P value [p = 0.891]). Effect estimates with overlapping confidence intervals. No evidence of opposing effects.

i. Serious imprecision. Confidence interval does not cross imprecision threshold (defined as 0.2*SD. This corresponds to 3 mmHg) but the optimal information size (OIS, defined as n = 400 following GRADE guidelines) was not met

j. Two out of the four studies included in the meta-analysis were fully or partially funded by private organizations or industries related to the berry market

k. Not important heterogeneity (I^2=0%, P value [p = 0.645]). Effect estimates with overlapping confidence intervals. No evidence of opposing effects.

l. Due to serious imprecision. Confidence interval crossed one imprecision threshold (defined as 0.2*SD . This corresponds to 2 mmHg). The optimal information size (OIS, defined as n = 400 following GRADE guidelines) was not met

References

1.Jeong, Han,Saem,Soon,Jun,Hong,Jae,Young,Cho,Tae-Bum,Lee,Ji-Wung,Kwon,Hyung,Joon,Joo,Jae,Hyoung,Park,Cheol,Woong,Yu,and,Do-Sun,Lim.. Effects of Rubus occidentalis extract on blood pressure in patients with prehypertension: Randomized, double-blinded, placebo-controlled clinical trial.Nutrition; 2016.

2.Richter, C. K.,A. C. Skulas-Ray,T. L. Gaugler,S. Meily,K. S. Petersen,and P. M. Kris-Etherton. Effects of Cranberry Juice Supplementation on Cardiovascular Disease Risk Factors in Adults with Elevated Blood Pressure: A Randomized Controlled Trial.Nutrients; 2021.

3.Le Sayec, M.,Y. Xu,M. Laiola,F. A. Gallego,D. Katsikioti,C. Durbidge,U. Kivisild,S. Armes,M. Lecomte,P. Fança-Berthon,E. Fromentin,F. Plaza Oñate,J. K. Cruickshank,and A. Rodriguez-Mateos. The effects of Aronia berry (poly)phenol supplementation on arterial function and the gut microbiome in middle aged men and women: Results from a randomized controlled trial.Clin Nutr; 2022.

4.Woolf, E. K.,J. D. Terwoord,N. S. Litwin,A. R. Vazquez,S. Y. Lee,N. Ghanem,K. A. Michell,B. T. Smith,L. E. Grabos,N. B. Ketelhut,N. P. Bachman,M. E. Smith,M. Le Sayec,S. Rao,C. L. Gentile,T. L. Weir,A. Rodriguez-Mateos,D. R. Seals,F. A. Dinenno,and S. A. Johnson.. Daily blueberry consumption for 12 weeks improves endothelial function in postmenopausal women with above-normal blood pressure through reductions in oxidative stress: a randomized controlled trial.Food Funct; 2023.

5.Johnson, Sarah A.,Arturo Figueroa,Negin Navaei,Alexei Wong,Roy Kalfon,Lauren T. Ormsbee,Rafaela G. Feresin,Marcus L. Elam,Shirin Hooshmand,Mark E. Payton,and Bahram H. Arjmandi. Daily Blueberry Consumption Improves Blood Pressure and Arterial Stiffness in Postmenopausal Women with Pre- and Stage 1-Hypertension: A Randomized, Double-Blind, Placebo-Controlled Clinical Trial.Journal of the Academy of Nutrition and Dietetics; 2015.

6.Keane, Karen M.,Trevor W. George,Costas L. Constantinou,Meghan A. Brown,Tom Clifford,and Glyn Howatson. Effects of Montmorency tart cherry (Prunus Cerasus L.) consumption on vascular function in men with early hypertension1.The American Journal of Clinical Nutrition; 2016.

7.Kianbakht, S.,and F. Hashem-Dabaghian. Antihypertensive efficacy and safety of Vaccinium arctostaphylos berry extract in overweight/obese hypertensive patients: A randomized, double-blind and placebo-controlled clinical trial.Complement Ther Med; 2019.

8.Tjelle, T. E.,L. Holtung,S. K. Bøhn,K. Aaby,M. Thoresen,SÅ Wiik,I. Paur,A. S. Karlsen,K. Retterstøl,P. O. Iversen,and R. Blomhoff. Polyphenol-rich juices reduce blood pressure measures in a randomised controlled trial in high normal and hypertensive volunteers.Br J Nutr; 2015.

9.Loo, B. M.,I. Erlund,R. Koli,P. Puukka,J. Hellström,K. Wähälä,P. Mattila,and A. Jula. Consumption of chokeberry (Aronia mitschurinii) products modestly lowered blood pressure and reduced low-grade inflammation in patients with mildly elevated blood pressure.Nutr Res; 2016.

Supplementary Table S6. GRADE evidence profile of additional outcomes (Vascular function markers, lipid profile, and inflammatory markers)

Certainty assessment							№ of patients		Effect		Certainty	Importance
№ of studies	Study design	Risk of bias	Inconsistency	Indirectness	Imprecision	Other considerations	Berry	Placebo	Relative (95% CI)	Absolute (95% CI)		
**Augmentation Index at 75 bpm (AIx@75) (follow-up: range 8 weeks to 12 weeks)**												
3[1,2,3]	randomised trials	not serious[a]	not serious[b]	not serious	serious[c]	publication bias strongly suspected[d]	91	89	-	MD **0.58 % lower** (2.93 lower to 1.78 higher)	⊕⊕◯◯ Low[a,b,c,d]	NOT IMPORTANT
**Flow-mediated dilation (FMD) (follow-up: 12 weeks)**												
2[1,2]	randomised trials	not serious[e]	serious[f]	not serious	very serious[g]	publication bias strongly suspected[d]	71	69	-	MD **0.41 % higher** (1.59 lower to 2.41 higher)	⊕◯◯◯ Very low[d,e,f,g]	NOT IMPORTANT
**Carotid-femoral pulse wave velocity (m/s) (follow-up: range 8 weeks to 12 weeks)**												
4[1,2,3,4]	randomised trials	not serious[h]	not serious[i]	not serious	serious[j]	publication bias strongly suspected[d]	111	109	-	MD **0.04 m/s higher** (0.14 lower to 0.21 higher)	⊕⊕◯◯ Low[d,h,i,j]	NOT IMPORTANT
**Brachial-aortic pulse wave velocity (m/s) (follow-up: 8 weeks)**												
2[4,5]	randomised trials	serious[k]	serious[l]	not serious	very serious[m]	publication bias strongly suspected[n]	50	35	-	MD **0.26 m/s lower** (1.51 lower to 0.99 higher)	⊕◯◯◯ Very low[k,l,m,n]	NOT IMPORTANT
**Total Cholesterol (mg/dL) (follow-up: range 8 weeks to 12 weeks)**												

Certainty assessment							№ of patients		Effect		Certainty	Importance
№ of studies	Study design	Risk of bias	Inconsistency	Indirectness	Imprecision	Other considerations	Berry	Placebo	Relative (95% CI)	Absolute (95% CI)		
4[1,2,3,6]	randomised trials	serious[o]	not serious[p]	not serious	serious[q]	publication bias strongly suspected[r]	109	108	-	MD **0.11 mg/dL higher** (4.58 lower to 4.8 higher)	⊕◯◯◯ Very low[o,p,q,r]	IMPORTANT
**LDL Cholesterol (mg/dL) (follow-up: range 8 weeks to 12 weeks)**												
3[1,2,3]	randomised trials	not serious[h]	not serious[s]	not serious	serious[t]	publication bias strongly suspected[d]	91	89	-	MD **2.51 mg/dL higher** (2.22 lower to 7.23 higher)	⊕⊕◯◯ Low[d,h,s,t]	IMPORTANT
**HDL Cholesterol (mg/dL) (follow-up: range 8 weeks to 12 weeks)**												
4[1,2,3,6]	randomised trials	serious[o]	not serious[u]	not serious	serious[v]	publication bias strongly suspected[r]	109	108	-	MD **0.49 mg/dL lower** (1.81 lower to 0.83 higher)	⊕◯◯◯ Very low[o,r,u,v]	IMPORTANT
**Tryglicerides (mg/dL) (follow-up: range 8 weeks to 12 weeks)**												
4[1,2,3,6]	randomised trials	serious[o]	serious[w]	not serious	serious[x]	publication bias strongly suspected[r]	109	108	-	MD **2.58 md/dL lower** (6.35 lower to 1.18 higher)	⊕◯◯◯ Very low[o,r,w,x]	IMPORTANT
**C Reactive Protein (mg/dL) (follow-up: range 8 weeks to 12 weeks)**												
3[3,5,7]	randomised trials	not serious[y]	not serious[z]	not serious	serious[aa]	publication bias strongly suspected[ab]	70	55	-	MD **0 mg/dL** (0.01 lower to 0.01 higher)	⊕⊕◯◯ Low[aa,ab,y,z]	NOT IMPORTANT
**TNF-alfa (pg/mL) (follow-up: 8 weeks)**												

Certainty assessment							№ of patients		Effect		Certainty	Importance
№ of studies	Study design	Risk of bias	Inconsistency	Indirectness	Imprecision	Other considerations	Berry	Placebo	Relative (95% CI)	Absolute (95% CI)		
2[6,7]	randomised trials	serious[ac,o]	very serious[ad]	not serious	very serious[ae]	publication bias strongly suspected[n]	38	39	-	MD **0.3 pg/mL higher** (1.76 lower to 2.36 higher)	⨁◯◯◯ Very low[ac,ad,ae,n,o]	NOT IMPORTANT

CI: confidence interval; **MD:** mean difference

Explanations

a. Included studies judged as low risk of bias correspond to more than 75% of weight in meta-analysis

b. Moderate heterogeneity (I^2=42.5%; P value [p = 0.175]). Effect estimates with overlapping confidence intervals. No evidence of opposing effects.

c. Serious imprecision. Confidence interval does not cross imprecision threshold (defined as 0.2*SD. This corresponds to 3%) but the optimal information size (OIS, defined as n = 400 following GRADE guidelines) was not met

d. All the included studies in the meta-analysis were fully or partially funded by private organizations or industries related to the berry market

e. Small number of studies included. However, the low risk of bias study included correspond to 60.9% of weight in meta-analysis

f. Substantial heterogeneity (I^2 = 73.3 %; P value [p = 0.053]) with opposite directions of effect across the two studies.

g. Very serious imprecision. Confidence interval crossed two imprecision thresholds (defined as 0.2*SD. This corresponds to 0.46%)

h. Included studies judged as low risk of bias correspond to more than 90% of weight in meta-analysis

i. Not important heterogeneity (I^2=0%; P value [p = 0.535]). Effect estimates with overlapping confidence intervals. No evidence of opposing effects.

j. Serious imprecision. Confidence interval does not cross imprecision threshold (defined as 0.2*SD. This corresponds to 0.6 m/s) but the optimal information size (OIS, defined as n = 400 following GRADE guidelines) was not met

k. Small number of studies included. The study judged as low risk of bias correspond to 35.1% of weight in meta-analysis. Point estimates direction differed between the low and the unclear risk of bias.

l. Moderate heterogeneity (I^2=44.9%; P value [p = 0.178]). The point estimates differed in direction between studies, suggesting true inconsistency. Small number of studies.

m. Very serious imprecision. Confidence interval crossed two imprecision thresholds (defined as 0.2*SD. This corresponds to 0.49 m/s)

n. Due to Johnson 2015 & Johnson 2017 was funded by private organizations related to the berry market

o. Due to unclear blinding in Loo, 2016 and lack of wash-out period lowering confidence in the estimated effect.

p. Not important heterogeneity (I^2=0%; P value [p = 0.809]). Effect estimates with overlapping confidence intervals. No evidence of opposing effects.

q. Serious imprecision. Confidence interval does not cross imprecision threshold (defined as 0.2*SD. This corresponds to 13 mg/dL) but the optimal information size (OIS, defined as n = 400 following GRADE guidelines) was not met

r. Three out of the four studies included in the meta-analysis were fully or partially funded by private organizations or industries related to the berry market

s. Not important heterogeneity (I^2=0%; P value [p = 0.458]). Effect estimates with overlapping confidence intervals. No evidence of opposing effects.

t. Serious imprecision. Confidence interval does not cross imprecision threshold (defined as 0.2*SD. This corresponds to 14 mg/dL) but the optimal information size (OIS, defined as n = 400 following GRADE guidelines) was not met

u. Not important heterogeneity (I^2=0%; P value [p = 0.768]). Effect estimates with overleaping confidence intervals. No evidence of opposing effects.

v. Serious imprecision. Confidence interval does not cross imprecision threshold (defined as 0.2*SD. This corresponds to 6 mg/dL) but the optimal information size (OIS, defined as n = 400 following GRADE guidelines) was not met

w. Not important heterogeneity (I^2=15.9%; P value [p = 0.312]). The point estimates differed in direction between studies, suggesting true inconsistency. Small number of studies.

x. Serious imprecision. Confidence interval does not cross imprecision threshold (defined as 0.2*SD. This corresponds to 13 mg/dL) but the optimal information size (OIS, defined as n = 400 following GRADE guidelines) was not met

y. Small number of studies included. The low risk of bias study included correspond to <50% of weight in meta-analysis. However, point estimates did not substantially differ between low and unclear risk of bias

z. Not important heterogeneity (I^2=0%; P value [p = 0.525]). Effect estimates with overleaping confidence intervals. No evidence of opposing effects.

aa. Serious imprecision. Confidence interval does not cross imprecision threshold (defined as 0.2*SD. This corresponds to 0.029 mg/dL) but the optimal information size (OIS, defined as n = 400 following GRADE guidelines) was not met

ab. Two out of the four included studies in the meta-analysis were fully or partially funded by private organizations or industries related to the berry market

ac. Small number of studies. Only unclear risk of bias studies available for meta-analysis.

ad. Considerable heterogeneity (I^2 = 89.4 %; P value [p = 0.002]) with opposite directions of effect across the two studies. Small number of studies.

ae. Very serious imprecision. Confidence interval crossed two imprecision thresholds (defined as 0.2*SD. This corresponds to 0.62 pg/mL)

References

1.Woolf, E. K.,J. D. Terwoord,N. S. Litwin,A. R. Vazquez,S. Y. Lee,N. Ghanem,K. A. Michell,B. T. Smith,L. E. Grabos,N. B. Ketelhut,N. P. Bachman,M. E. Smith,M. Le Sayec,S. Rao,C. L. Gentile,T. L. Weir,A. Rodriguez-Mateos,D. R. Seals,F. A. Dinenno,and S. A. Johnson.. Daily blueberry consumption for 12 weeks improves endothelial function in postmenopausal women with above-normal blood pressure through reductions in oxidative stress: a randomized controlled trial.Food Funct; 2023.

2.Le Sayec, M.,Y. Xu,M. Laiola,F. A. Gallego,D. Katsikioti,C. Durbidge,U. Kivisild,S. Armes,M. Lecomte,P. Fança-Berthon,E. Fromentin,F. Plaza Oñate,J. K. Cruickshank,and A. Rodriguez-Mateos. The effects of Aronia berry (poly)phenol supplementation on arterial function and the gut microbiome in middle aged men and women: Results from a randomized controlled trial.Clin Nutr; 2022.

3.Richter, C. K.,A. C. Skulas-Ray,T. L. Gaugler,S. Meily,K. S. Petersen,and P. M. Kris-Etherton. Effects of Cranberry Juice Supplementation on Cardiovascular Disease Risk Factors in Adults with Elevated Blood Pressure: A Randomized Controlled Trial.Nutrients; 2021.

4.Johnson, Sarah A.,Arturo Figueroa,Negin Navaei,Alexei Wong,Roy Kalfon,Lauren T. Ormsbee,Rafaela G. Feresin,Marcus L. Elam,Shirin Hooshmand,Mark E. Payton,and Bahram H. Arjmandi. Daily Blueberry Consumption Improves Blood Pressure and Arterial Stiffness in Postmenopausal Women with Pre- and Stage 1-Hypertension: A Randomized, Double-Blind, Placebo-Controlled Clinical Trial.Journal of the Academy of Nutrition and Dietetics; 2015.

5.Jeong, Han,Saem,Soon,Jun,Hong,Jae,Young,Cho,Tae-Bum,Lee,Ji-Wung,Kwon,Hyung,Joon,Joo,Jae,Hyoung,Park,Cheol,Woong,Yu,and,Do-Sun,Lim.. Effects of Rubus occidentalis extract on blood pressure in patients with prehypertension: Randomized, double-blinded, placebo-controlled clinical trial.Nutrition; 2016.

6.Loo, B. M.,I. Erlund,R. Koli,P. Puukka,J. Hellström,K. Wähälä,P. Mattila,and A. Jula. Consumption of chokeberry (Aronia mitschurinii) products modestly lowered blood pressure and reduced low-grade inflammation in patients with mildly elevated blood pressure.Nutr Res; 2016.

7.Johnson, S. A.,R. G. Feresin,N. Navaei,A. Figueroa,M. L. Elam,N. S. Akhavan,S. Hooshmand,S. Pourafshar,M. E. Payton,and B. H. Arjmandi. Effects of daily blueberry consumption on circulating biomarkers of oxidative stress, inflammation, and antioxidant defense in postmenopausal women with pre- and stage 1-hypertension: a randomized controlled trial.Food Funct; 2017.

Supplementary Table S7. PRISMA 2020 Checklist.

Section and Topic	Item #	Checklist item	Location where item is reported
**TITLE**			
Title	1	Identify the report as a systematic review.	1
**ABSTRACT**			
Abstract	2	See the PRISMA 2020 for Abstracts checklist.	1
**INTRODUCTION**			
Rationale	3	Describe the rationale for the review in the context of existing knowledge.	2
Objectives	4	Provide an explicit statement of the objective(s) or question(s) the review addresses.	2-3
**METHODS**			
Eligibility criteria	5	Specify the inclusion and exclusion criteria for the review and how studies were grouped for the syntheses.	3
Information sources	6	Specify all databases, registers, websites, organisations, reference lists and other sources searched or consulted to identify studies. Specify the date when each source was last searched or consulted.	4
Search strategy	7	Present the full search strategies for all databases, registers and websites, including any filters and limits used.	4 and Supplementary Material
Selection process	8	Specify the methods used to decide whether a study met the inclusion criteria of the review, including how many reviewers screened each record and each report retrieved, whether they worked independently, and if applicable, details of automation tools used in the process.	4
Data collection process	9	Specify the methods used to collect data from reports, including how many reviewers collected data from each report, whether they worked independently, any processes for obtaining or confirming data from study investigators, and if applicable, details of automation tools used in the process.	4-5
Data items	10a	List and define all outcomes for which data were sought. Specify whether all results that were compatible with each outcome domain in each study were sought (e.g. for all measures, time points, analyses), and if not, the methods used to decide which results to collect.	3-4
	10b	List and define all other variables for which data were sought (e.g. participant and intervention characteristics, funding sources). Describe any assumptions made about any missing or unclear information.	4
Study risk of bias assessment	11	Specify the methods used to assess risk of bias in the included studies, including details of the tool(s) used, how many reviewers assessed each study and whether they worked independently, and if applicable, details of automation tools used in the process.	6
Effect measures	12	Specify for each outcome the effect measure(s) (e.g. risk ratio, mean difference) used in the synthesis or presentation of results.	4-5
Synthesis methods	13a	Describe the processes used to decide which studies were eligible for each synthesis (e.g. tabulating the study intervention characteristics and comparing against the planned groups for each synthesis (item #5)).	4-5

Section and Topic	Item #	Checklist item	Location where item is reported
	13b	Describe any methods required to prepare the data for presentation or synthesis, such as handling of missing summary statistics, or data conversions.	5-6
	13c	Describe any methods used to tabulate or visually display results of individual studies and syntheses.	6
	13d	Describe any methods used to synthesize results and provide a rationale for the choice(s). If meta-analysis was performed, describe the model(s), method(s) to identify the presence and extent of statistical heterogeneity, and software package(s) used.	5-6
	13e	Describe any methods used to explore possible causes of heterogeneity among study results (e.g. subgroup analysis, meta-regression).	5-6
	13f	Describe any sensitivity analyses conducted to assess robustness of the synthesized results.	5-6
Reporting bias assessment	14	Describe any methods used to assess risk of bias due to missing results in a synthesis (arising from reporting biases).	6
Certainty assessment	15	Describe any methods used to assess certainty (or confidence) in the body of evidence for an outcome.	6
**RESULTS**			
Study selection	16a	Describe the results of the search and selection process, from the number of records identified in the search to the number of studies included in the review, ideally using a flow diagram.	6-7
	16b	Cite studies that might appear to meet the inclusion criteria, but which were excluded, and explain why they were excluded.	6 and Supplementary Material
Study characteristics	17	Cite each included study and present its characteristics.	6
Risk of bias in studies	18	Present assessments of risk of bias for each included study.	11-12
Results of individual studies	19	For all outcomes, present, for each study: (a) summary statistics for each group (where appropriate) and (b) an effect estimate and its precision (e.g. confidence/credible interval), ideally using structured tables or plots.	12-13
Results of syntheses	20a	For each synthesis, briefly summarise the characteristics and risk of bias among contributing studies.	13
	20b	Present results of all statistical syntheses conducted. If meta-analysis was done, present for each the summary estimate and its precision (e.g. confidence/credible interval) and measures of statistical heterogeneity. If comparing groups, describe the direction of the effect.	13-32
	20c	Present results of all investigations of possible causes of heterogeneity among study results.	33-35
	20d	Present results of all sensitivity analyses conducted to assess the robustness of the synthesized results.	36-37
Reporting biases	21	Present assessments of risk of bias due to missing results (arising from reporting biases) for each synthesis assessed.	27

Section and Topic	Item #	Checklist item	Location where item is reported
Certainty of evidence	22	Present assessments of certainty (or confidence) in the body of evidence for each outcome assessed.	27-32
**DISCUSSION**			
Discussion	23a	Provide a general interpretation of the results in the context of other evidence.	33-34
	23b	Discuss any limitations of the evidence included in the review.	35
	23c	Discuss any limitations of the review processes used.	35
	23d	Discuss implications of the results for practice, policy, and future research.	35-36
**OTHER INFORMATION**			
Registration and protocol	24a	Provide registration information for the review, including register name and registration number, or state that the review was not registered.	1,3
	24b	Indicate where the review protocol can be accessed, or state that a protocol was not prepared.	3
	24c	Describe and explain any amendments to information provided at registration or in the protocol.	NA
Support	25	Describe sources of financial or non-financial support for the review, and the role of the funders or sponsors in the review.	37
Competing interests	26	Declare any competing interests of review authors.	37
Availability of data, code and other materials	27	Report which of the following are publicly available and where they can be found: template data collection forms; data extracted from included studies; data used for all analyses; analytic code; any other materials used in the review.	36-37

From: Page MJ, McKenzie JE, Bossuyt PM, Boutron I, Hoffmann TC, Mulrow CD, et al. The PRISMA 2020 statement: an updated guideline for reporting systematic reviews. BMJ 2021;372:n71. doi: 10.1136/bmj.n71. This work is licensed under CC BY 4.0. To view a copy of this license, visit https://creativecommons.org/licenses/by/4.0/
